# Supplementary material for: An Aurora B-RPA signaling axis secures chromosome segregation fidelity
Source: Nat Commun. 2023 May 25;14:3008. doi: 10.1038/s41467-023-38711-2 (PMC10212944; doi:10.1038/s41467-023-38711-2)
Supplement: Supplementary file 1 — Supplementary Information [file 41467_2023_38711_MOESM1_ESM.docx]

**Supplementary Information**

**An Aurora B-RPA signaling axis secures chromosome segregation fidelity**

Poonam Roshan^1, #^, Sahiti Kuppa^2, #^, Jenna R. Mattice^3^, Vikas Kaushik^2^, Rahul Chadda^2^, Nilisha Pokhrel^3^, Brunda R. Tumala^2^, Aparna Biswas^1^, Brian Bothner^3^, Edwin Antony^2, *^ and Sofia Origanti^1, *^

**
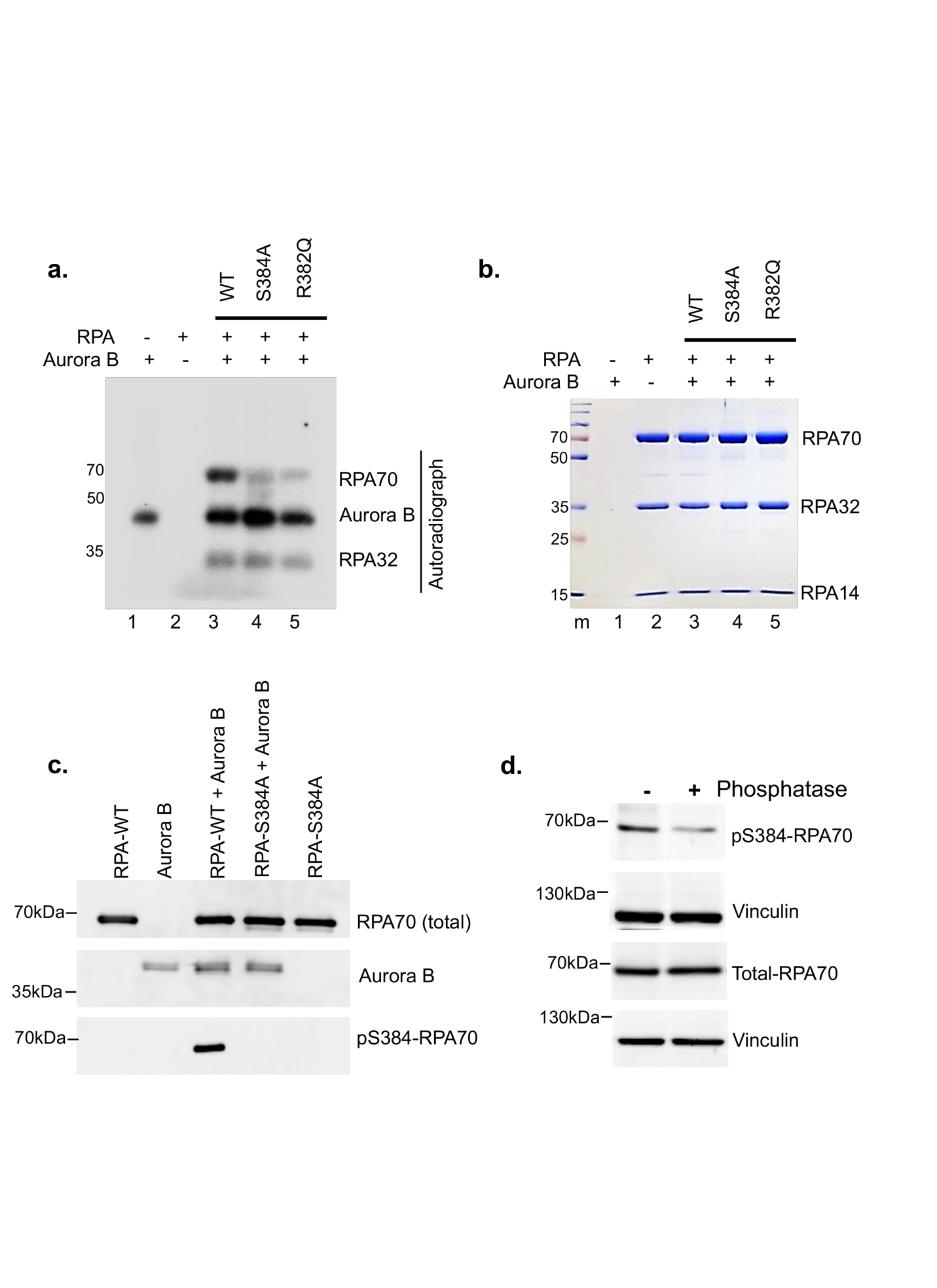
**

**Supplementary Figure 1. *In vitro* kinase assay shows site-specific phosphorylation of RPA70 on Ser-384 by Aurora B**. **a)** Representative autoradiograph shows Aurora B kinase-dependent phosphorylation of RPA70 that is lost upon phosphosite-Ser384 to Alanine substitution or phospho-motif Arg-382 to Gln cancer-specific mutation. Recombinant human RPA was incubated with Aurora B kinase and subjected to *in vitro* kinase assay using γ-p^32^-ATP. Autophosphorylation of Aurora B kinase is also shown as control. Blots are representative of three independent experiments. **b)** Samples corresponding to **a.** were subjected to SDS-PAGE analysis and Coomassie stained to show equal concentrations of WT-RPA and mutants assayed per reaction. Gels are representative of three independent experiments. m=molecular weight standard. **c**) Western blots represent cold i*n vitro* kinase reactions that were assayed with the indicated antibodies and depict the specificity of the Ser-384 RPA70 phospho-specific antibody. **d**) HCT116 cell lysates were treated with and without phosphatase and assayed by western blotting to depict specificity of the phospho Ser-384 RPA70 antibody. All gels and blots shown in this figure are representative of three independent experiments.

**
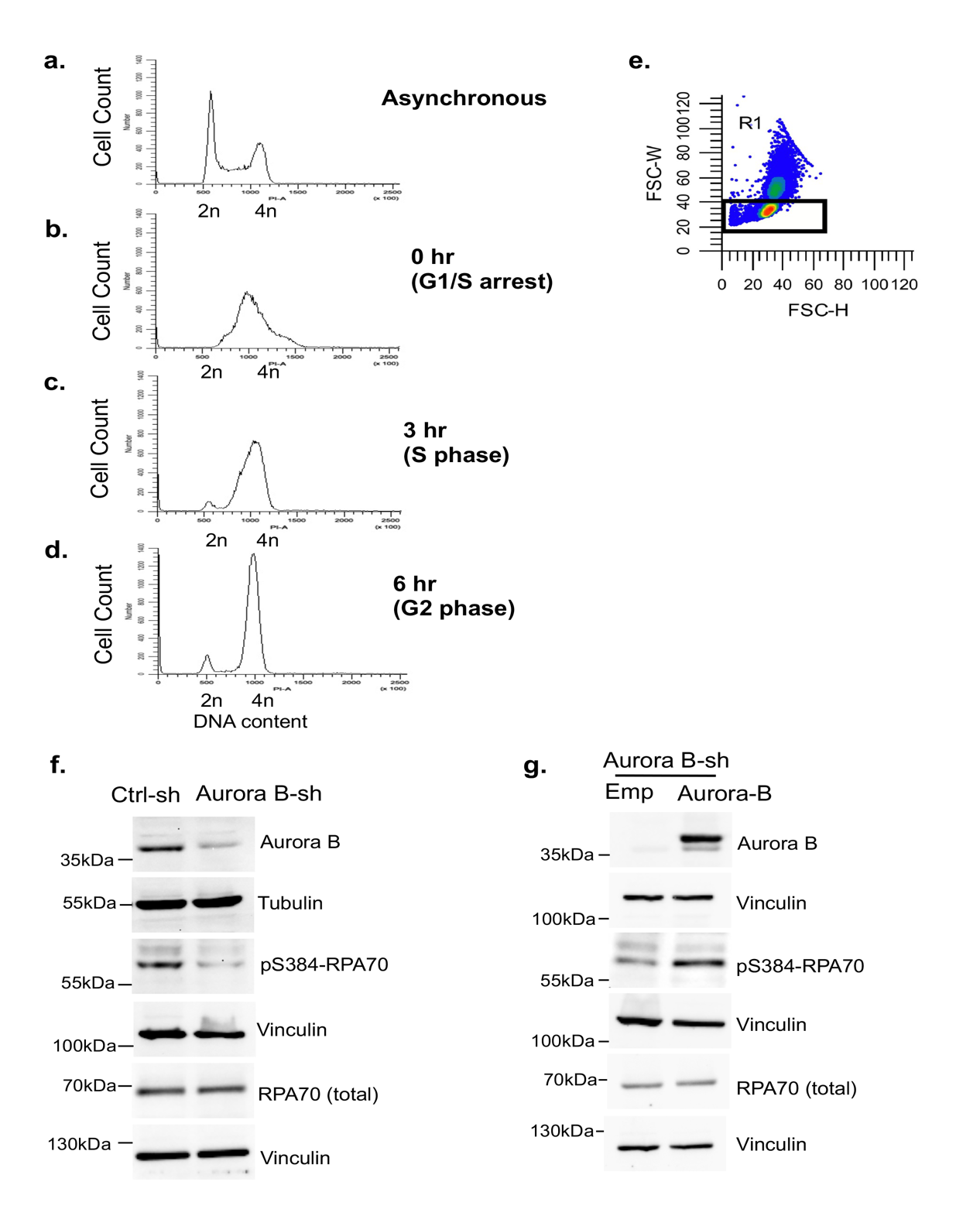
**

**Supplementary Figure 2. Cell synchronization at G1/S boundary using double thymidine block and decrease in S384-RPA70 phosphorylation by knockdown of Aurora B. a-d)** Cell cycle profile represents synchronization of parental HCT116 cells at the G1/S boundary (**b**) using double-thymidine block followed by progression into S phase at 3 hours (**c**) and into G2 phase at 6 hours after release from double-thymidine block (**d**). Asynchronous cells (**a**) used as controls. DNA content was analyzed using propidium iodide staining and flow cytometry. Plots are representative of three independent experiments. **e)** The plot shown exemplifies the gating strategy used for all FACS data. A singlet gate was created by plotting FSC-H versus FSC-W to identify the single cell cluster. Cells showing similar FSC-H signal, but high FSC-W signal were excluded from this gate due to the high probability of doublets. After establishing the singlet gate, there were no more gates set. The ModFit LT software analyzed the single cells/nuclei using the algorithm based on the diploid model selected to determine the percentage of cells in each part of the cycle. **f**) Western blots represent Aurora B knockdown using shRNA and a corresponding decrease in phospho S384-RPA70 levels in mitotic cells with no changes in total RPA70 levels. Anti-Vinculin and anti-Tubulin antibodies were used as loading control. **g**) Blots represent the rescue of Aurora B levels in cells knocked down for Aurora B and a corresponding rescue of phospho S384-RPA70 levels relative to the empty vector control (Emp) in mitotic cells. All blots are representative of three independent experiments.

**
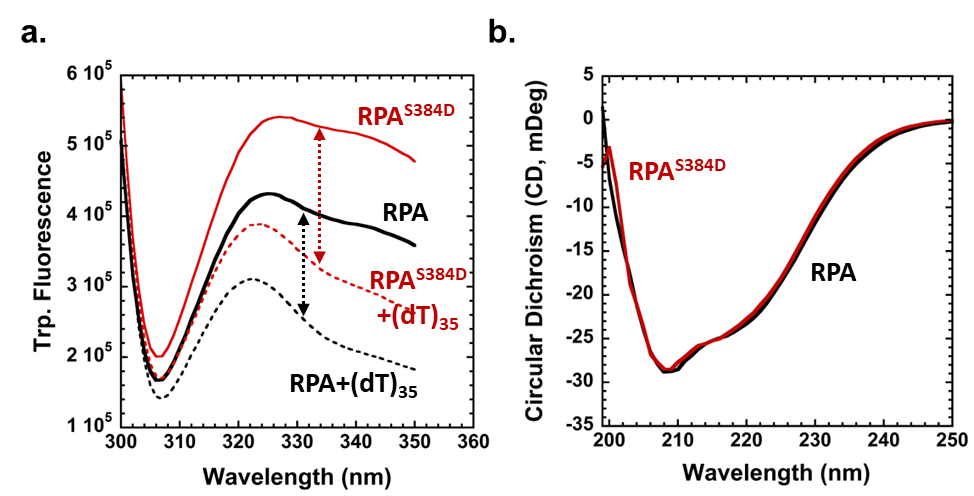
**

**Supplementary Figure 3. Configurational changes in RPA induced by Ser-384 phosphorylation. a)** Changes in intrinsic tryptophan (Trp) fluorescence were collected by exciting RPA or RPA^S384D^ at 295 nm in the presence or absence of (dT)_35_ ssDNA. RPA^S384D^ shows a ~50% enhancement in intrinsic Trp fluorescence compared to RPA. These data show that the domains undergo a configurational change in the phosphomimetic RPA protein leading to a change in the local environment around the Trp residues. Upon ssDNA binding, both RPA and RPA^S384D^ show a similar degree of quenching of Trp fluorescence suggesting no major perturbations in the ssDNA binding properties. **b)** Circular dichroism (CD) analysis of RPA or RPA^S384D^ show no major changes in the overall secondary structures within the domains suggesting that the phosphomimetic substitution does not alter the secondary structure of DBD-B or the other domains in RPA.

**
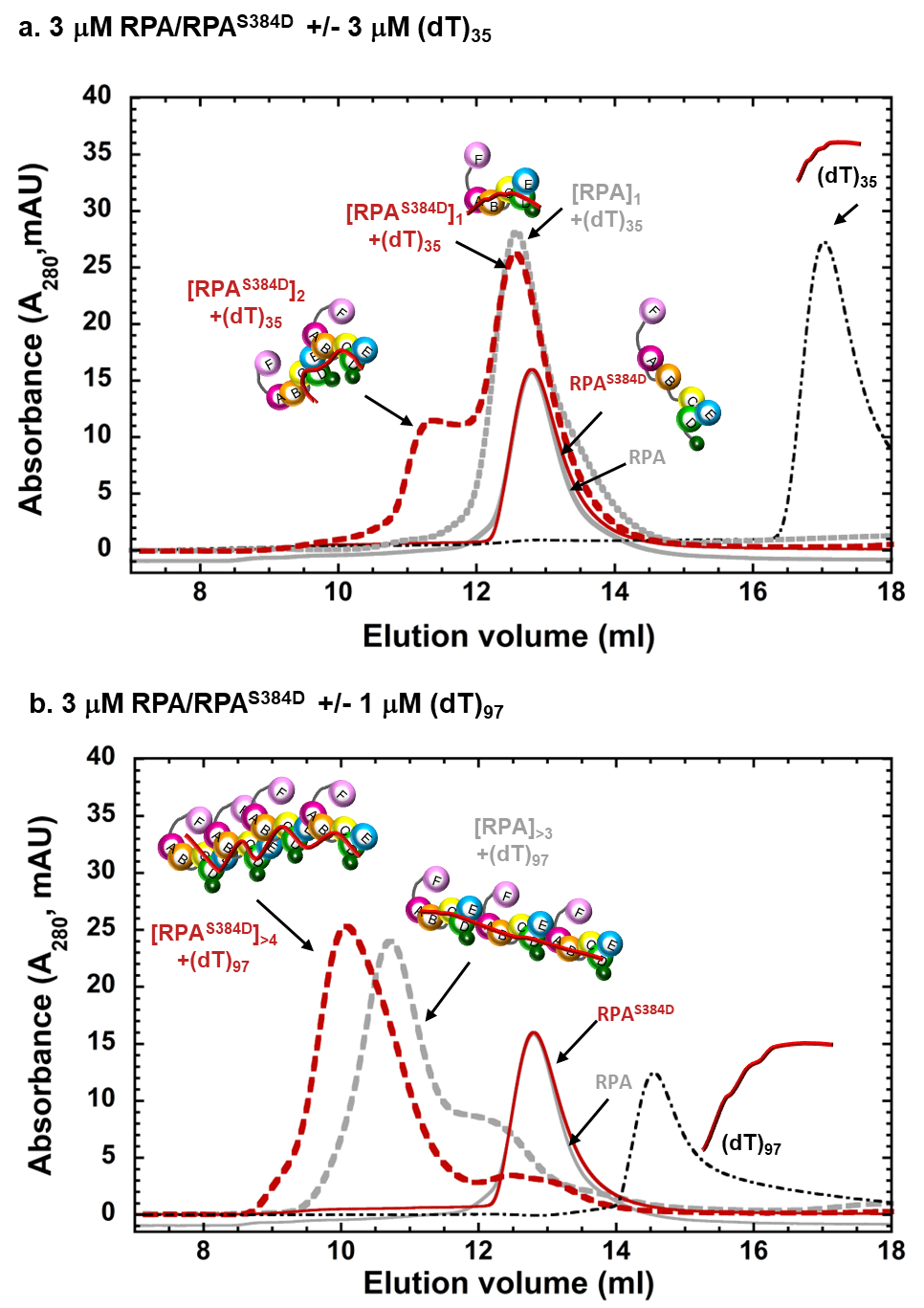
**

**Supplementary Figure 4. Ser-384 phosphorylation promotes formation of higher density RPA-ssDNA nucleoprotein filaments.** RPA binding to **a)** short (dT)_35_ or **b)** long (dT)_97_ ssDNA substrates were assessed by size exclusion chromatography (SEC). RPA and the RPA^S384D^ phosphomimetic elute as single peaks in the absence of ssDNA. On (dT)_35_, incubation of equimolar concentrations of RPA and ssDNA results in a single peak for RPA suggesting formation of a 1:1 complex. However, for RPA^S384D^, a major 1:1 peak is observed along with a minor (larger) 2:1 (RPA^S384D^:DNA) complex peak. This phenomenon is exaggerated on the longer (dT)_97_ substrates where a higher molar ratio of RPA:DNA is used. Here, RPA predominantly forms a 3:1 complex whereas RPA^S384D^ forms a much larger complex which is likely composed of ~4 or 5 RPA^S384D^ bound per (dT)_97_.

**
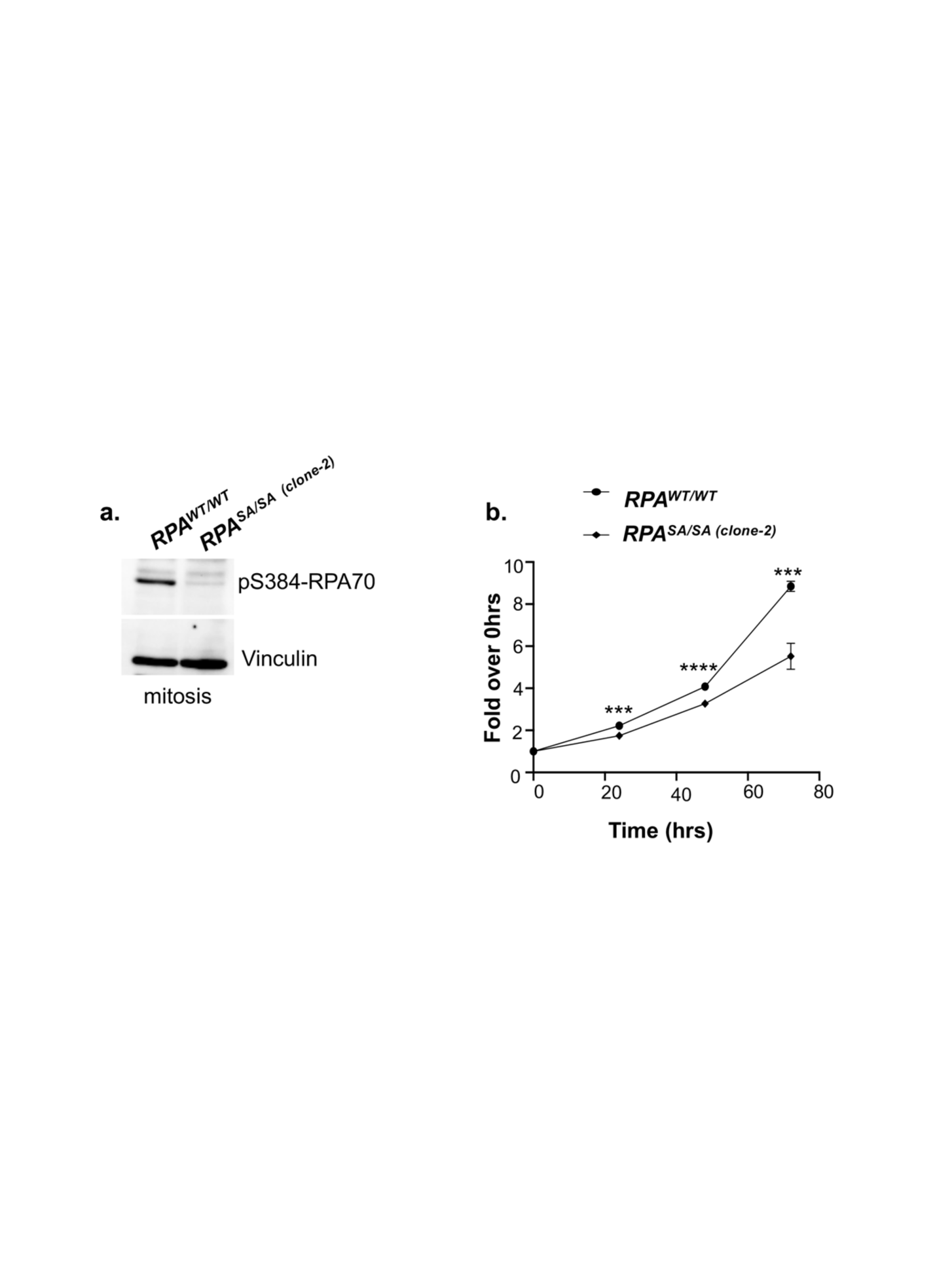
**

**Supplementary Figure 5. Homozygous knock-in RPA^SA/SA^ mutant clone#2 also exhibits marked loss of viability. a)** Representative western blot depicts loss of Ser-384 RPA70 phosphorylation in the second clone of RPA^SA/SA^ mutant cells synchronized in mitosis. Blot is representative of three independent experiments. **b)** MTS assay shows decreased viability of phospho-dead *RPA^SA/SA^* mutant. Cells were assayed at 0, 24, 48 and 72 hours of growth. Values corrected for background absorbance were normalized to 0 hrs of growth. Error=SEM. Mean of three independent experiments are plotted. Triplicate wells were assayed per time point for each experiment. Statistical significance was determined using an unpaired two-tailed *t*-test: ****p*=0.0004 at 24 hours, *****p*=0.000023 at 48 hours, and ****p*=0.00013 at 72 hours.

**
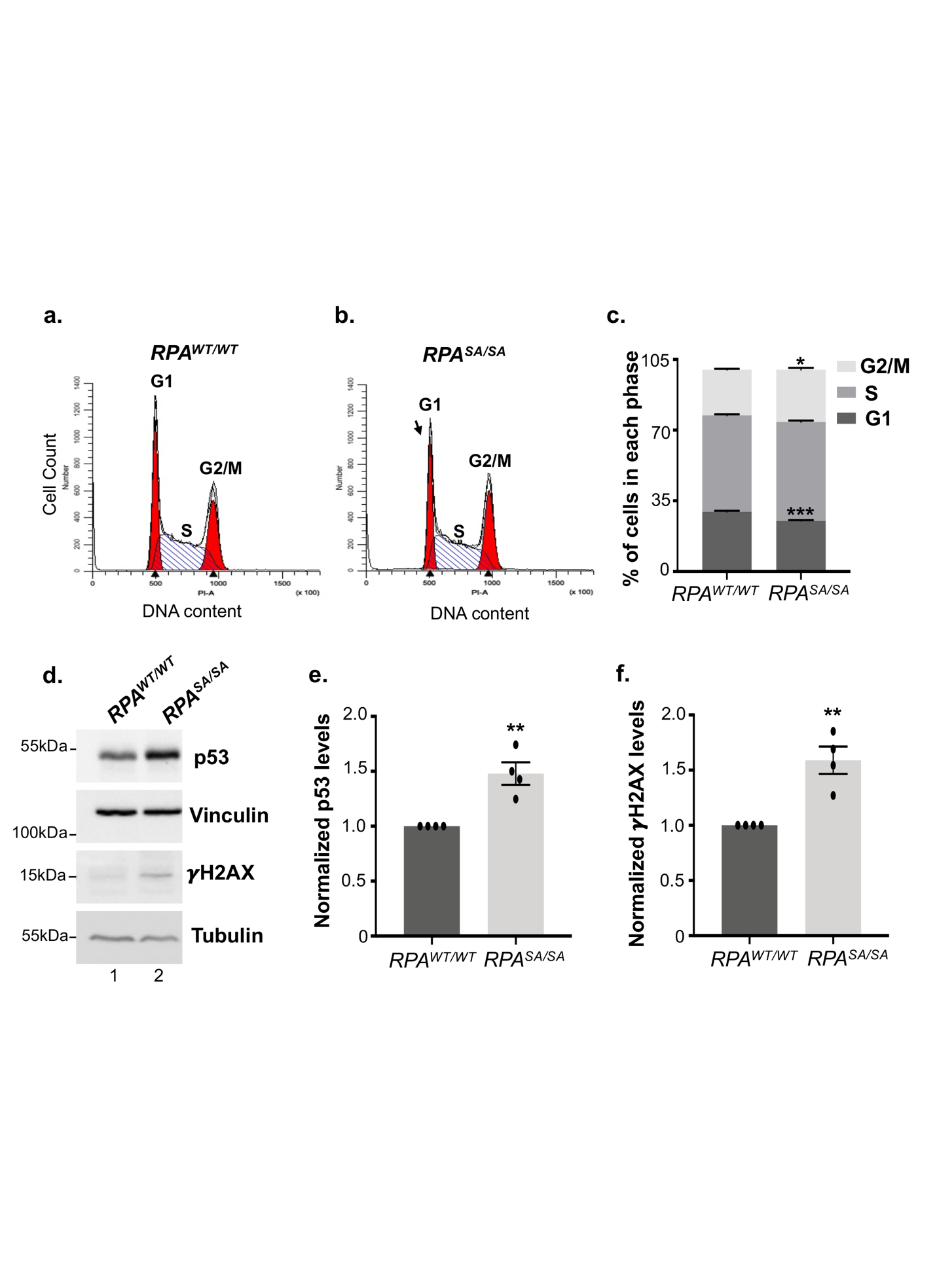
**

**Supplementary Figure 6. Mild decrease in percentage of cells in G1 phase of cell cycle in the *RPA^SA/SA^* mutant cells.** Cell cycle profile of asynchronous *RPA^WT/WT^* (**a**) and *RPA^SA/SA^* mutant (**b**) cells were analyzed by flow cytometry and DNA content was assessed by propidium iodide staining. Profiles are representative of three independent experiments. **c**) The profiles shown in **a.** and **b.** were quantitated and mean cell percentages in each phase of cell cycle were plotted. Bar graph shows mean of three independent experiments. Error = SEM. Statistical significance was determined using an unpaired two-tailed *t*-test: ****p*=0.0003 and **p*=0.0245. **d.** Western blot represents basal genomic stress response in *RPA^SA/SA^* mutant. Blots were probed with the indicated antibodies and are representative of three independent experiments. **e)** and **f)** Plots depict the quantitation of basal p53 and 𝜸H2AX levels respectively in WT relative to *RPA^SA/SA^* mutant from 4 different experiments. p53 and 𝜸H2AX levels were normalized to their respective loading controls. Error=SEM. Statistical significance was determined using an unpaired two-tailed *t*-test: ***p=*0.0034 (p53) and *p=*0.0031(𝜸H2AX).

**
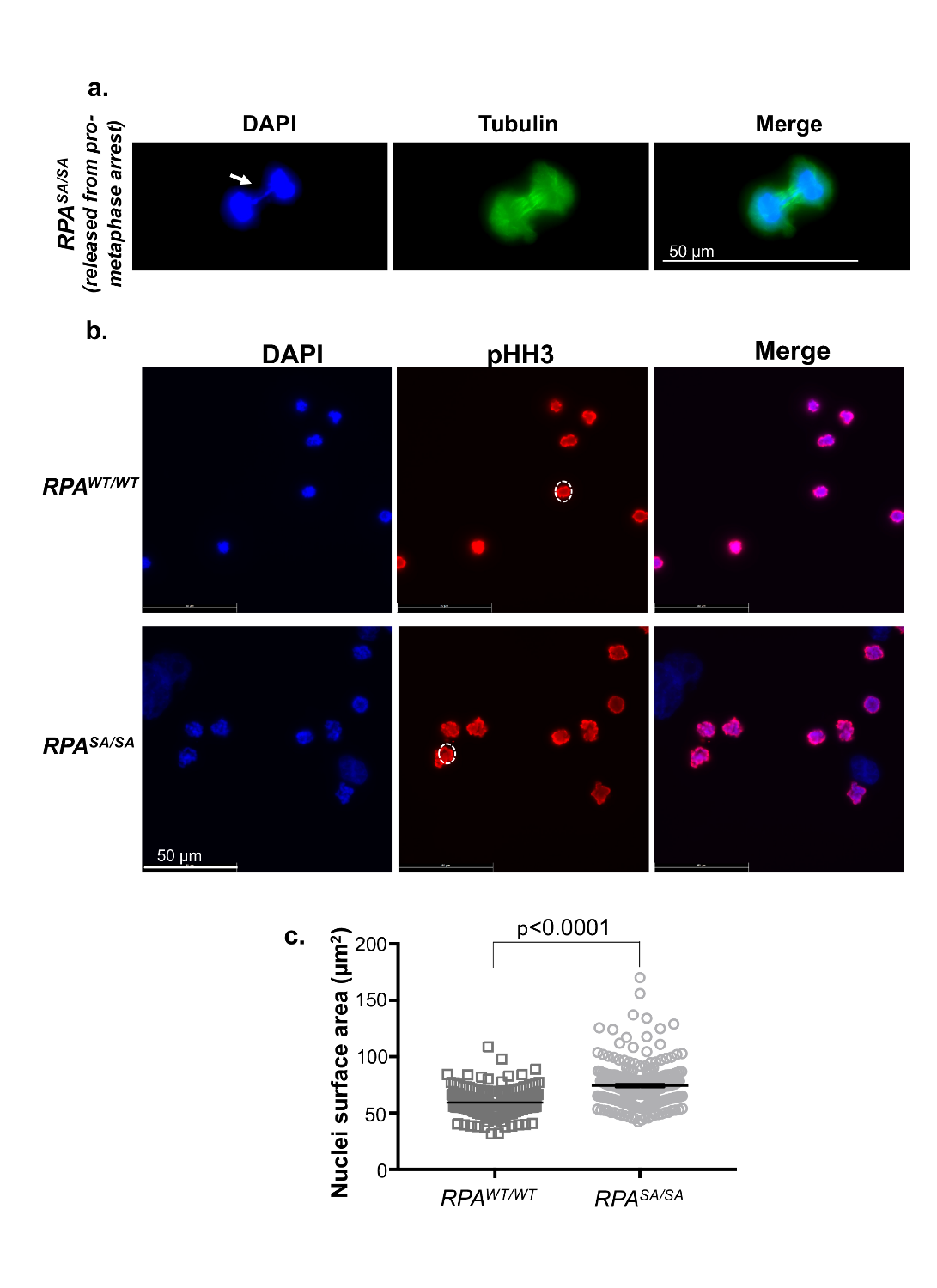
**

**Supplementary Figure 7. Defects in chromosome segregation and condensation induced by loss of Ser384-RPA70 phosphorylation. a)** Representative immunofluorescent images stained with DAPI, and anti-Tubulin antibody depict anaphase bridges (white arrow) in cells released from prometaphase arrest. Images are representative of three independent experiments. **b)** Representative immunofluorescent images stained with DAPI and anti-phoshpho-Ser10-Histone H3 antibody depict less chromosome condensation (white dotted circles) in *RPA^SA/SA^* mutant cells arrested in prometaphase. Images are representative of three independent experiments.

**
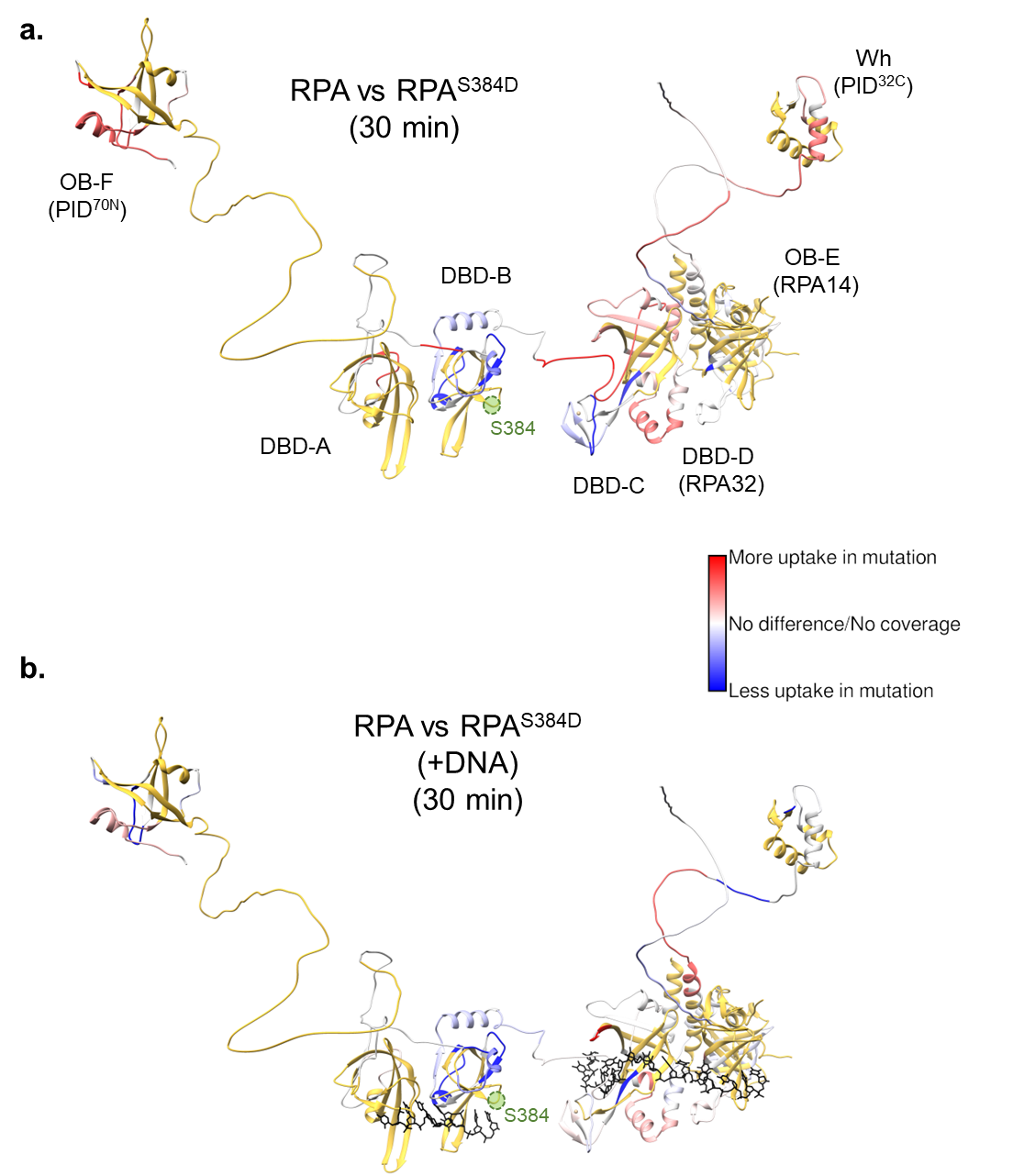
**

**Supplementary Figure 8. Configurational changes in RPA are induced by a S384D substitution within the Aurora kinase motif in DBD-B.** HDX changes between RPA and RPA^S384D^ are shown in the **a)** absence or **b)** presence of ssDNA. Changes in deuterium uptake/loss are observed in almost all DNA binding and protein-interaction domains. Data are mapped onto the structure of human RPA which is built using the structures of the OB domains from crystal structures. The regions colored yellow corresponds to peptides that were not identified in the MS analysis of either or both the wild type and mutant RPA samples. The flexible linkers were modeled using AlphaFold. Position of Ser-384 is denoted in green. Dataset from the 30 min timepoint are shown. Data are presented as +/- SDM from three independent experiments.

**
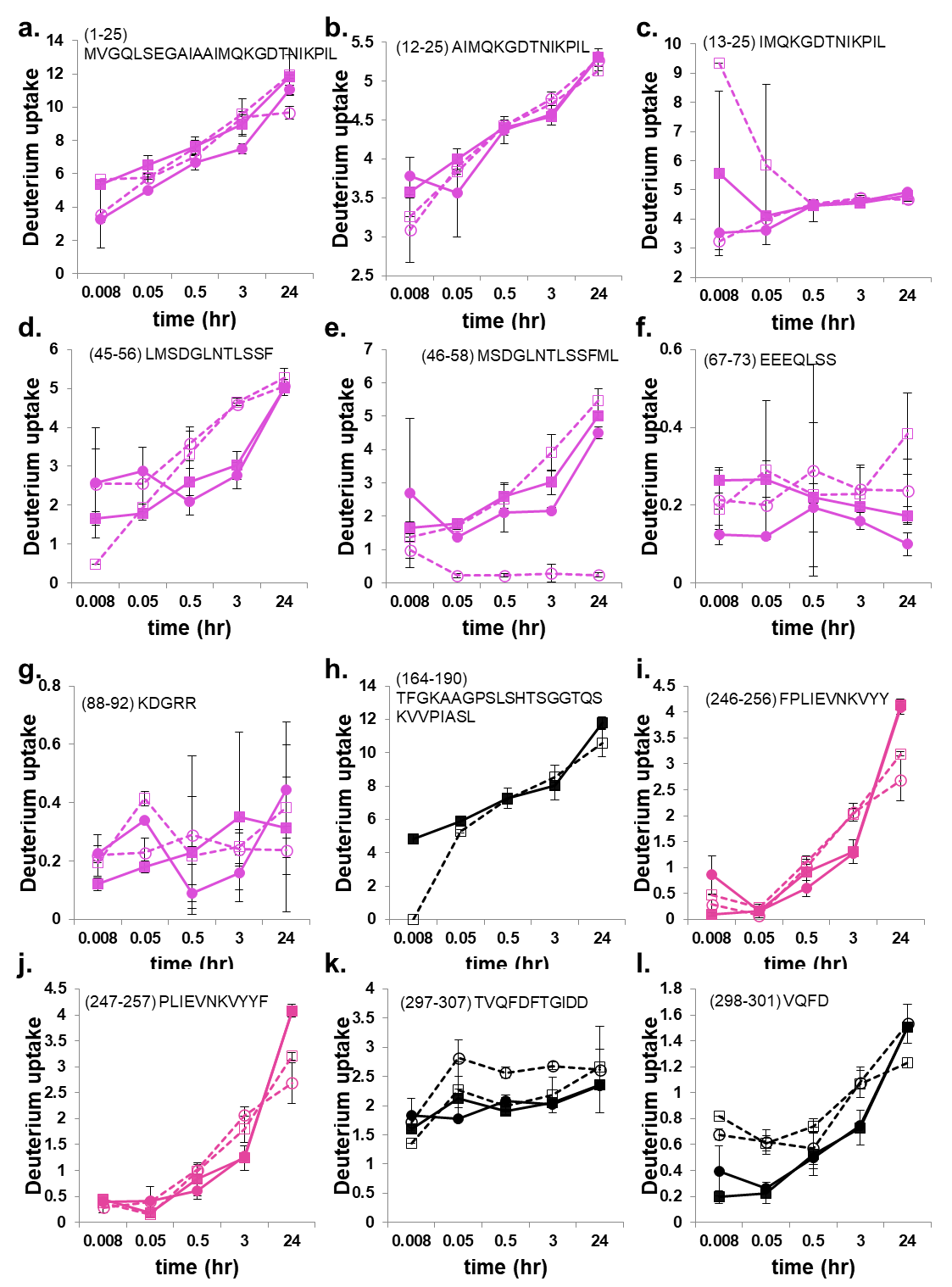
**

**Supplementary Figure 9. HDX-MS analysis of RPA and RPA^S384D^ peptides from RPA70 in the absence or presence of ssDNA.** HDX-MS data corresponding to specific peptides from RPA and RPA^S384D^ are shown for samples measured in the absence (solid lines) or presence of ssDNA ((dT)_35_)) (dotted lines). Symbols denote RPA (●), RPA+DNA (○), RPA^S384D^ (■), and RPA^S384D^+DNA (□). Peptides from OB-F or PID^70N^ are shown in violet and DBD-A are shown in pink. The data in black are peptides from the F-A and A-B linkers. The amino acid residue numbers and sequence of the corresponding peptides are noted. Data are presented as +/- SDM from three independent experiments.

**
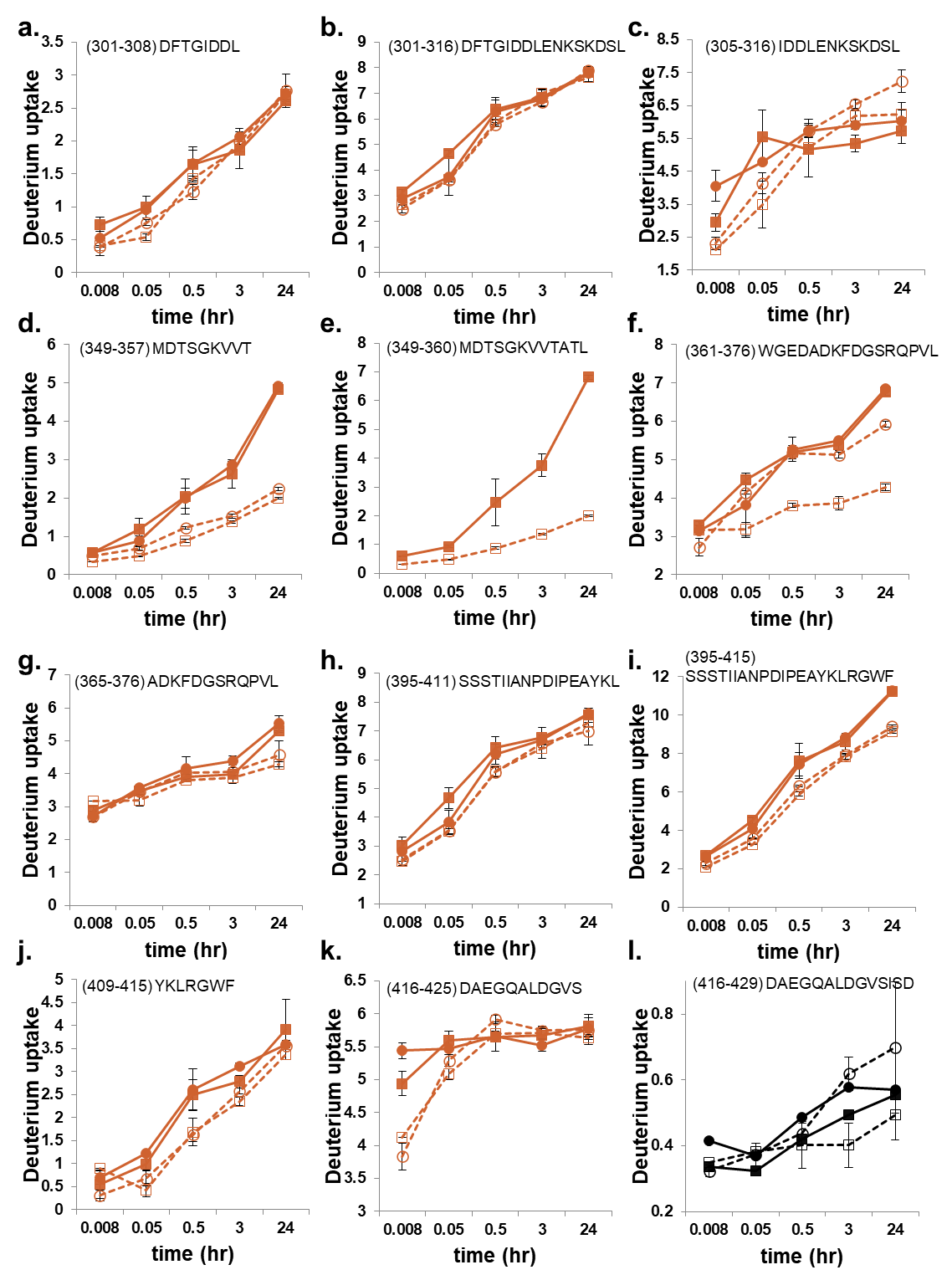
**

**Supplementary Figure 10. HDX-MS analysis of RPA and RPA^S384D^ peptides from RPA70 in the absence or presence of ssDNA.** HDX-MS data corresponding to specific peptides from RPA and RPA^S384D^ are shown for samples measured in the absence (solid lines) or presence of ssDNA ((dT)_35_)) (dotted lines). Symbols denote RPA (●), RPA+DNA (○), RPA^S384D^ (■), and RPA^S384D^+DNA (□). Peptides from DBD-B are shown in orange. The data in black are peptides from the B-C linker. The amino acid residue numbers and sequence of the corresponding peptides are noted. Data are presented as +/- SDM from three independent experiments.

**
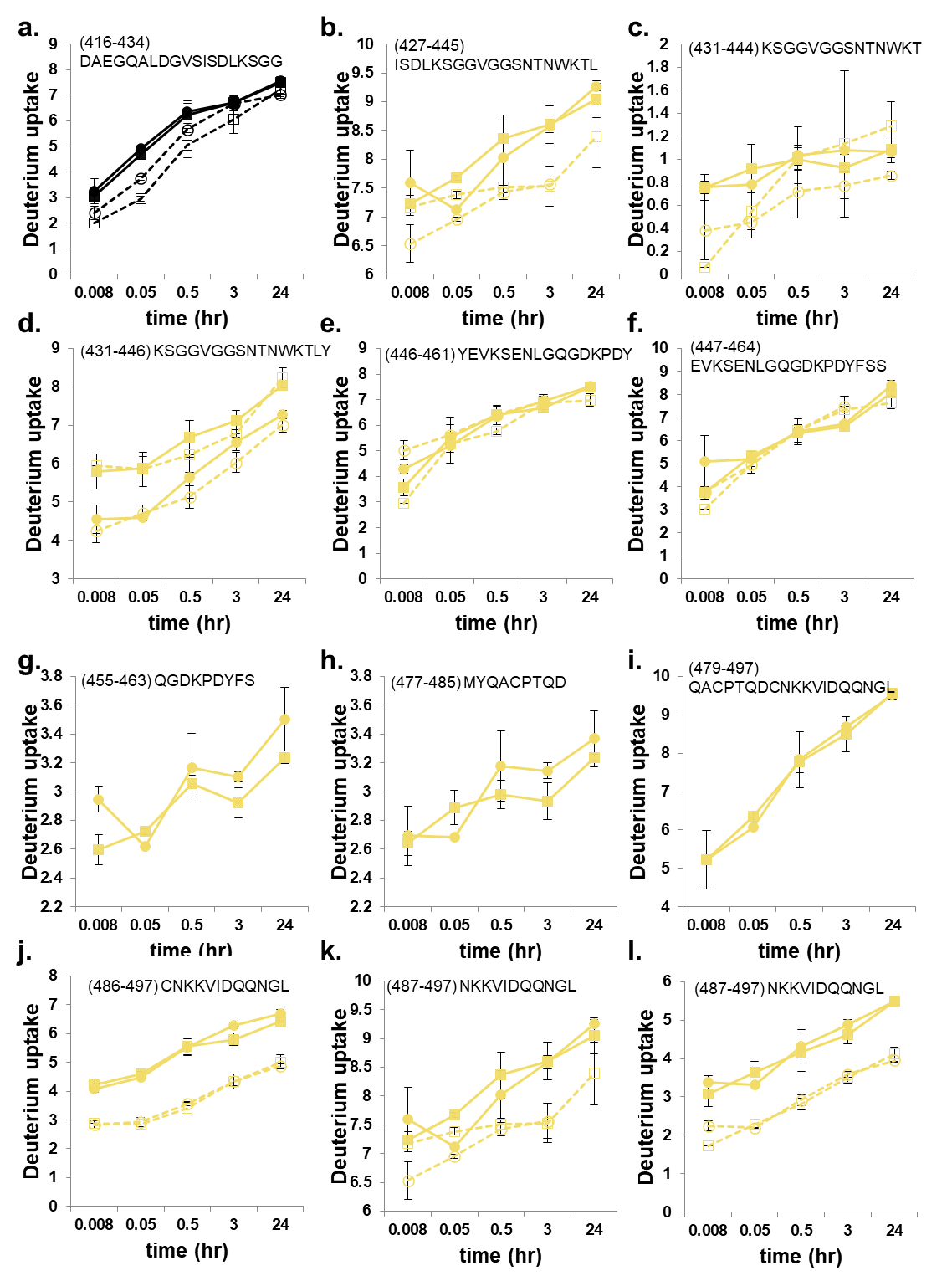
**

**Supplementary Figure 11. HDX-MS analysis of RPA and RPA^S384D^ peptides from RPA70 in the absence or presence of ssDNA.** HDX-MS data corresponding to specific peptides from RPA and RPA^S384D^ are shown for samples measured in the absence (solid lines) or presence of ssDNA ((dT)_35_)) (dotted lines). Symbols denote RPA (●), RPA+DNA (○), RPA^S384D^ (■), and RPA^S384D^+DNA (□). Peptides from DBD-C are shown in yellow. The data in black are peptides from the B-C linker. The amino acid residue numbers and sequence of the corresponding peptides are noted. Data are presented as +/- SDM from three independent experiments.

**
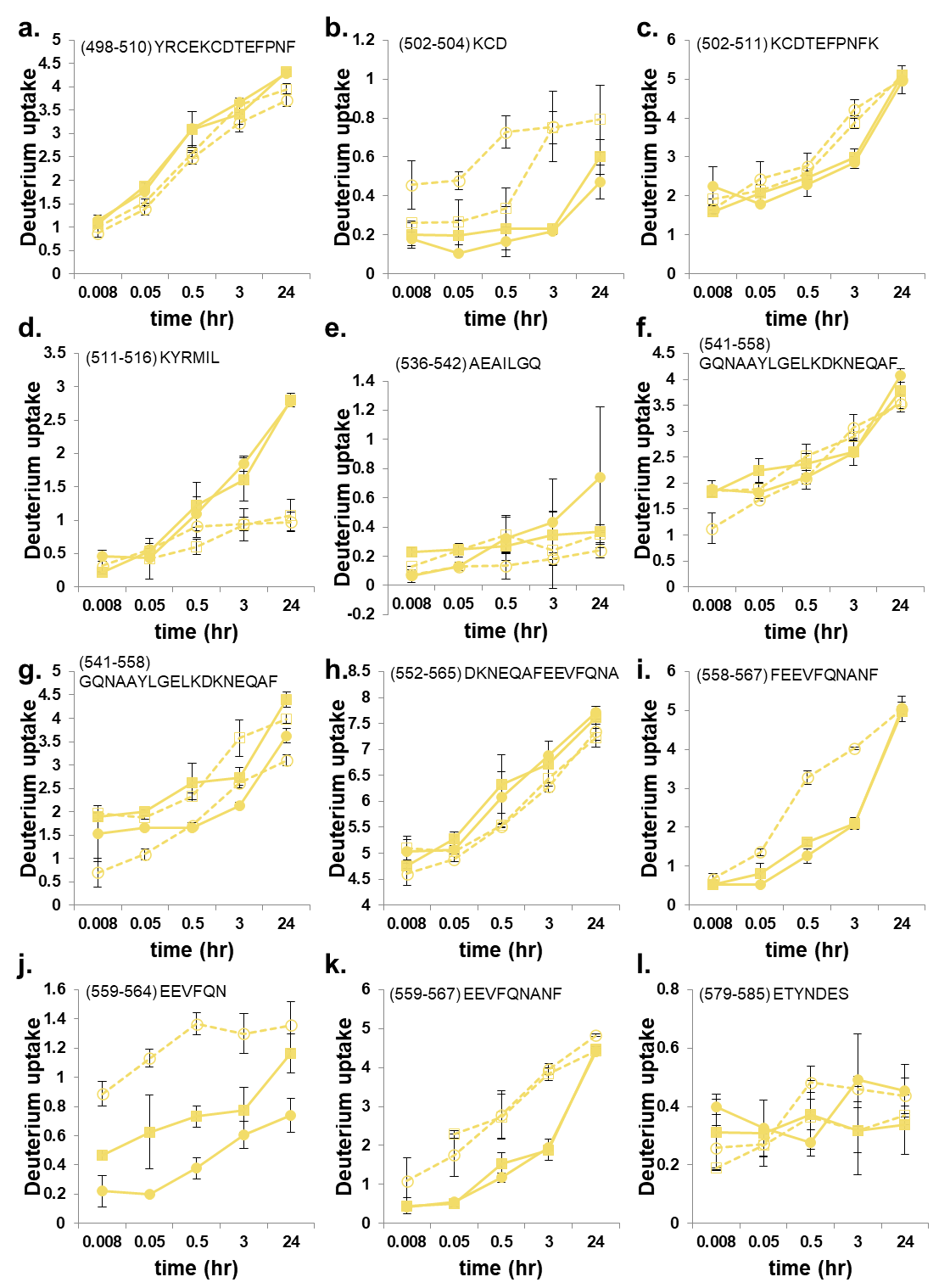
**

**Supplementary Figure 12. HDX-MS analysis of RPA and RPA^S384D^ peptides from RPA70 in the absence or presence of ssDNA.** HDX-MS data corresponding to specific peptides from RPA and RPA^S384D^ are shown for samples measured in the absence (solid lines) or presence of ssDNA ((dT)_35_)) (dotted lines). Symbols denote RPA (●), RPA+DNA (○), RPA^S384D^ (■), and RPA^S384D^+DNA (□). Peptides from DBD-C are shown in yellow. The amino acid residue numbers and sequence of the corresponding peptides are noted. Data are presented as +/- SDM from three independent experiments.

**
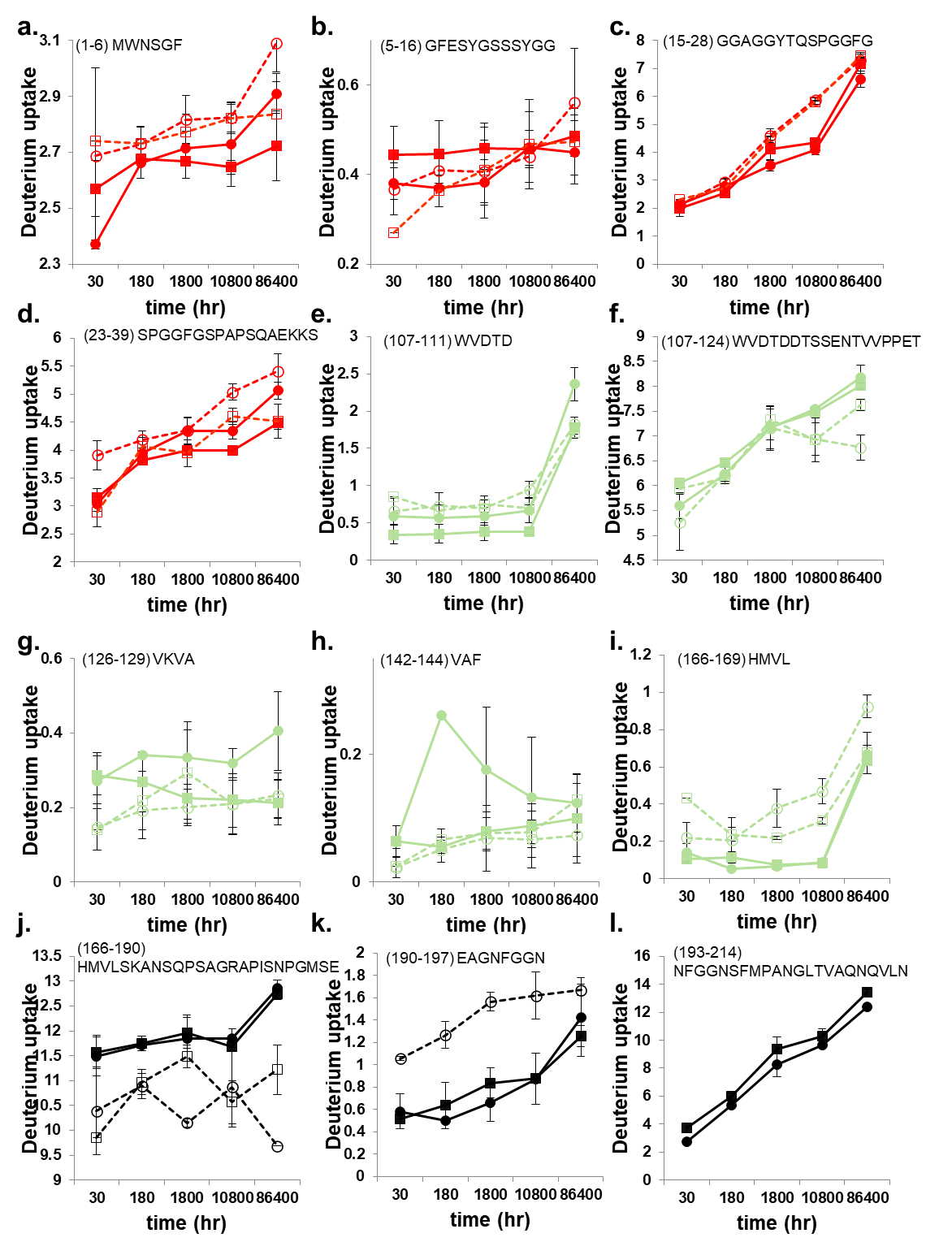
**

**Supplementary Figure 13. HDX-MS analysis of RPA and RPA^S384D^ peptides from RPA32 in the absence or presence of ssDNA.** HDX-MS data corresponding to specific peptides from RPA and RPA^S384D^ are shown for samples measured in the absence (solid lines) or presence of ssDNA ((dT)_35_)) (dotted lines). Symbols denote RPA (●), RPA+DNA (○), RPA^S384D^ (■), and RPA^S384D^+DNA (□). Peptides from the N-terminal hyperphosphorylation region are denoted in red and peptides from DBD-D are shown in green. The data in black are peptides from the D-wh linker. The amino acid residue numbers and sequence of the corresponding peptides are noted. Data are presented as +/- SDM from three independent experiments.

**
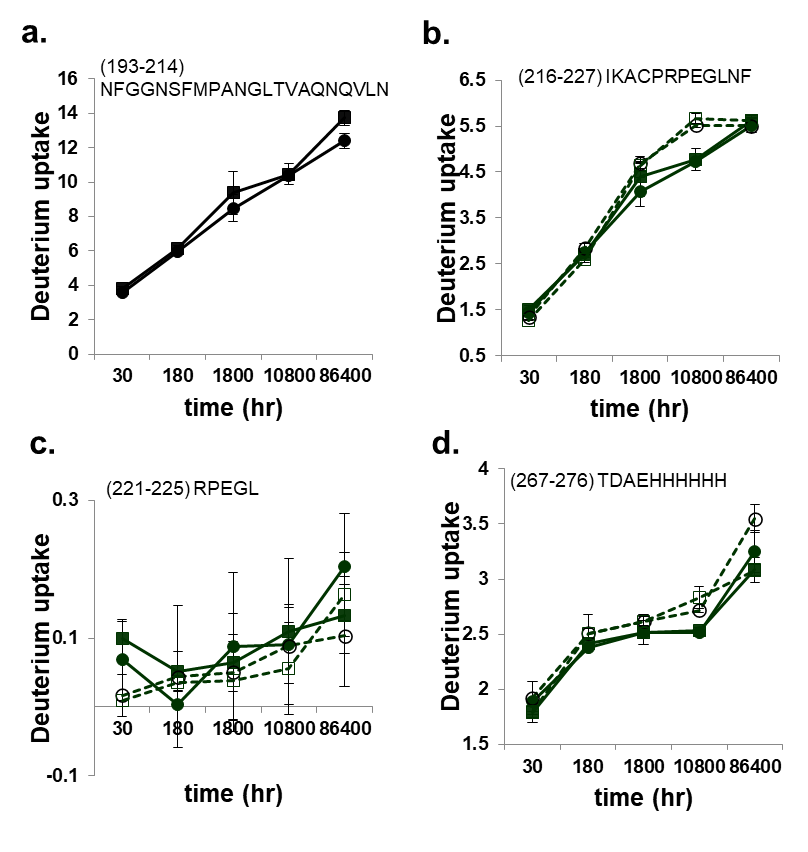
**

**Supplementary Figure 14. HDX-MS analysis of RPA and RPA^S384D^ peptides from RPA32 in the absence or presence of ssDNA.** HDX-MS data corresponding to specific peptides from RPA and RPA^S384D^ are shown for samples measured in the absence (solid lines) or presence of ssDNA ((dT)_35_)) (dotted lines). Symbols denote RPA (●), RPA+DNA (○), RPA^S384D^ (■), and RPA^S384D^+DNA (□). Peptides from the winged helix (wh or PID^32C^) are shown in dark green. The data in black are peptides from the D-wh linker. The amino acid residue numbers and sequence of the corresponding peptides are noted. Data are presented as +/- SDM from three independent experiments.

**
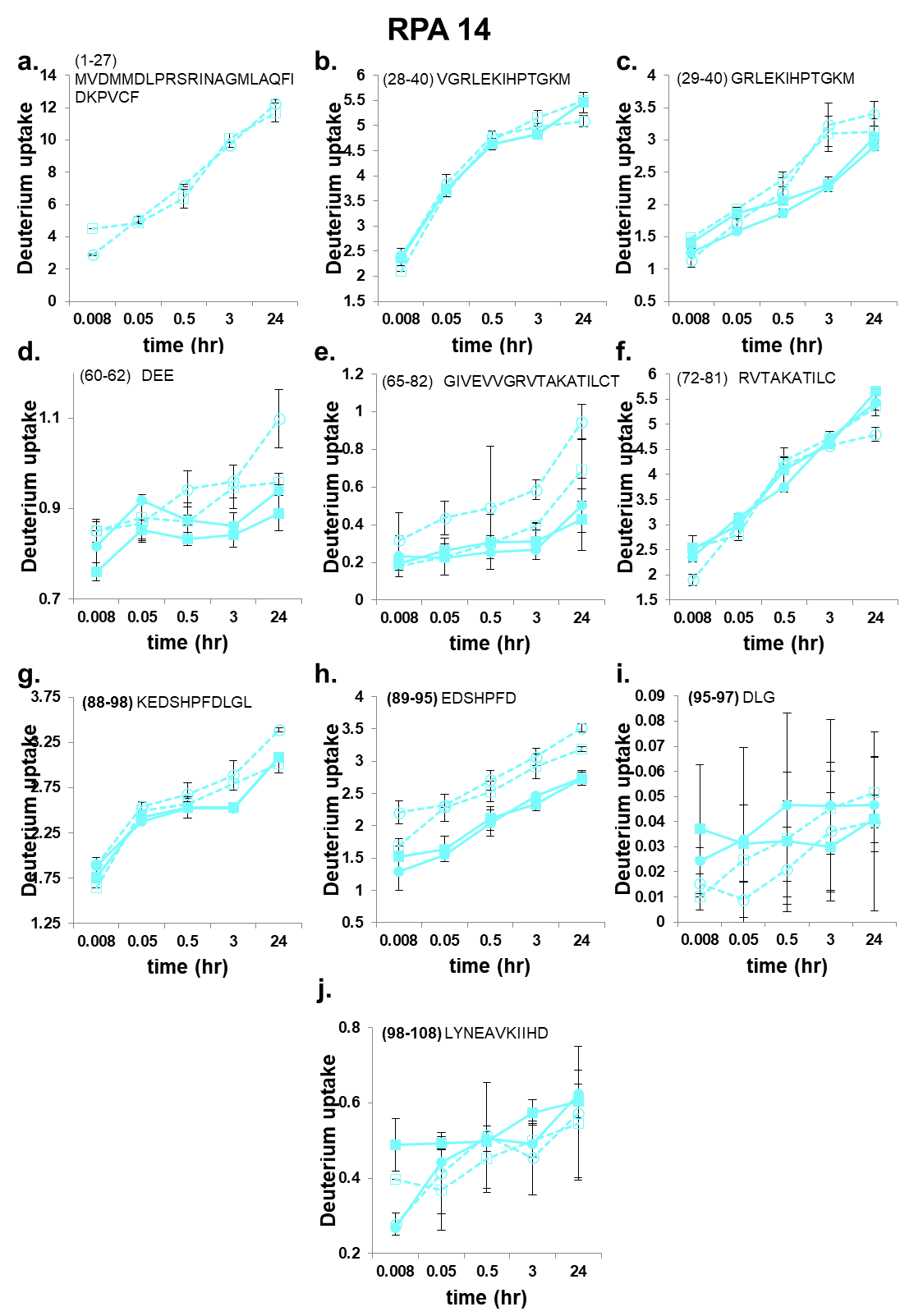
**

**Supplementary Figure 15. HDX-MS analysis of RPA and RPA^S384D^ peptides from RPA14 in the absence or presence of ssDNA.** HDX-MS data corresponding to specific peptides from RPA and RPA^S384D^ are shown for samples measured in the absence (solid lines) or presence of ssDNA ((dT)_35_)) (dotted lines). Symbols denote RPA (●), RPA+DNA (○), RPA^S384D^ (■), and RPA^S384D^+DNA (□). Peptides from RPA14 are shown in cyan. The amino acid residue numbers and sequence of the corresponding peptides are noted. Data are presented as +/- SDM from three independent experiments.


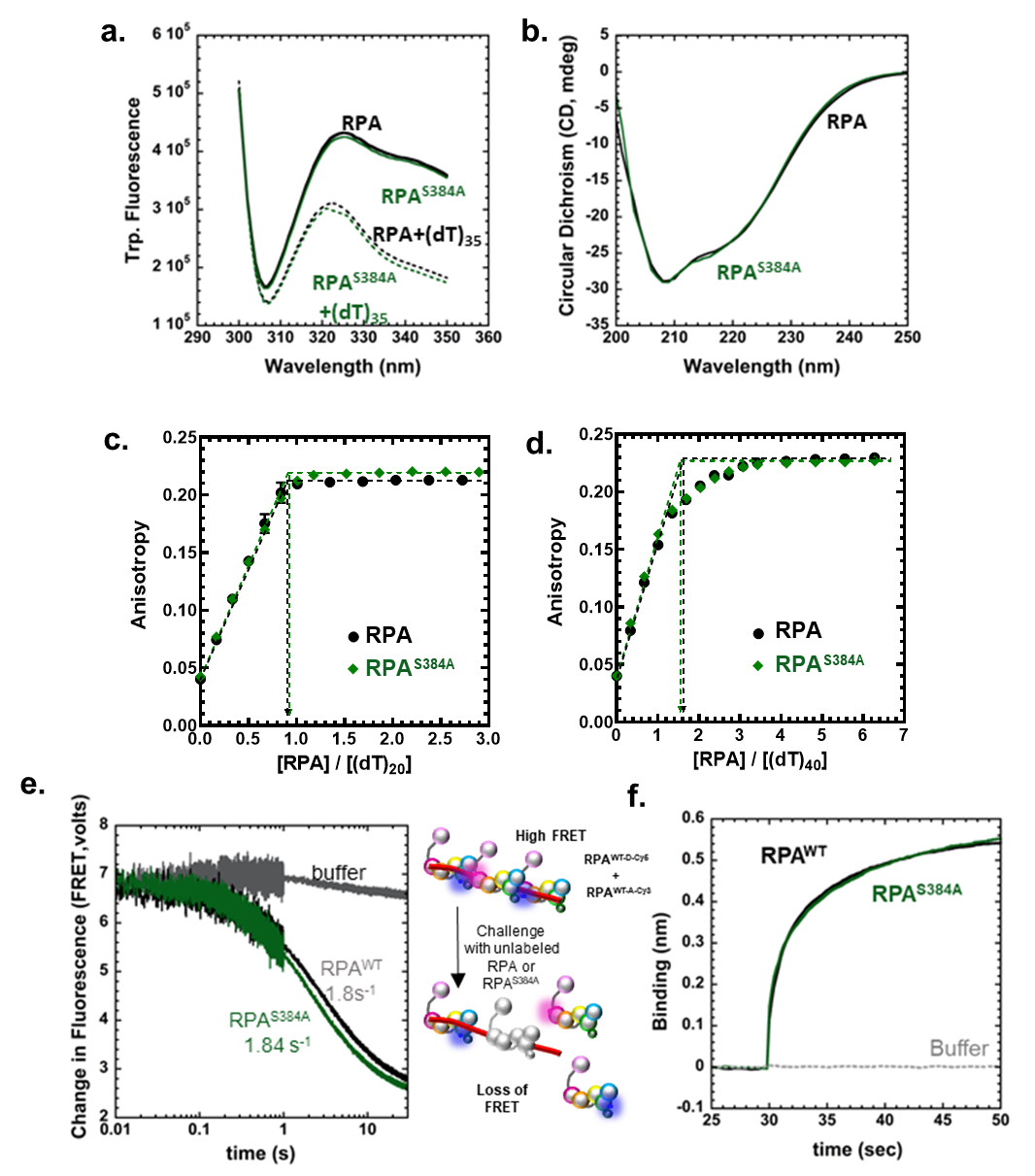


**Supplementary Figure 16. RPA carrying a Ser-384 to Ala substitution behaves similar to wildtype RPA.** RPA and RPA^S384A^ were compared for various biochemical activities and show very similar activities. **a)** Similar intrinsic Trp fluorescence profiles and quenching upon ssDNA binding. **b)** Secondary structures as measured by circular dichroism were also similar. ssDNA binding to **c)** short or **d)** longer ssDNA were also similar. For all data, SE from three independent experiments are shown.**e)** In stopped flow facilitated exchange experiments both proteins remodeled fluorescent RPA bound to ssDNA with similar kinetics and efficiency. Errors reported are +/- SEM from three independent experiments. **f)** Finally, both proteins bound with similar affinity profiles to DSS1 in biolayer interferometry analysis in the absence of ssDNA.

**Supplementary Table 1.**

| **HDX data summary** | |
| --- | --- |
| Protein State | WT, WT+ssDNA, S384D, S384D+ssDNA |
| HDX reaction details | diluted 1:10 into deuterated reaction buffer (30 mM HEPES, 200 mM KCl, pD 7.8) at 22°C |
| Time course | 0, 0.008, 0.05, 0.5, 3, 30 h |
| Controls | diluted into a non-deuterated reaction buffer  (30 mM HEPES, 200 mM KCl, pD 7.8 at 22°C) |
| Number of peptides | RPA14 = 11 peptides  RPA32 = 18 peptides  RPA70 = 47 peptides |
| Sequence Coverage | RPA14 = 68% coverage  RPA32 = 50% coverage  RPA70 = 52% coverage |
| Average peptide length/ redundancy | RPA14: average peptide length = 10.5, redundancy = 0.115  RPA32: average peptide length = 9.4, redundancy = 0.06  RPA70: average peptide length = 12.3, redundancy = 0.07 |
| Experimental Replicates | n=3 for each condition and time point |
| Repeatability | The average standard deviation from experimental replicates of the deuterium content calculated for each time point and condition |
| Total exchange reported (as percentage) | RPA14 = 44 % exchange  RPA32 = 42 % exchange  RPA70 = 48% |
| Coefficient of variation for 24h time point averaged | RPA14 = 0.112  RPA32 = 0.122  RPA70 = 0.069 |

**Supplementary Raw Images**

**
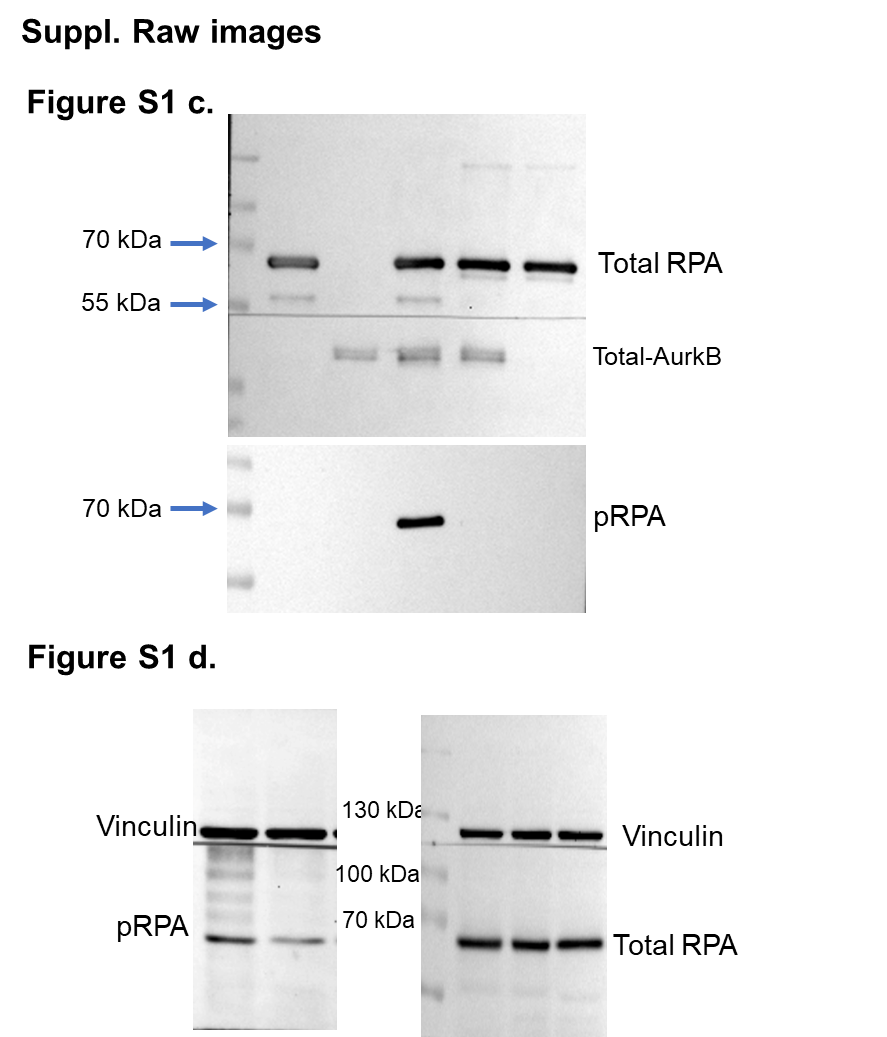
**

**
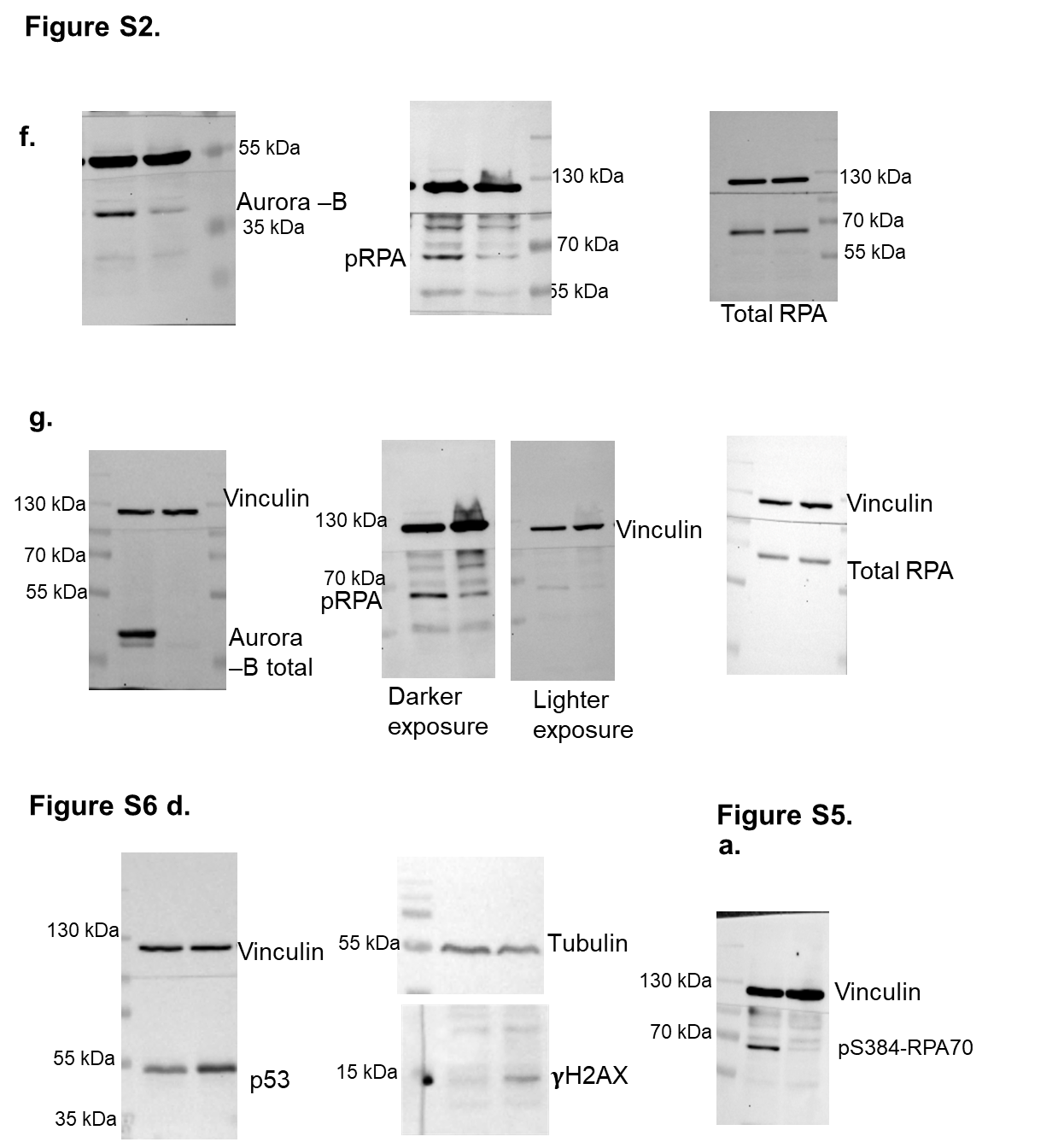
**
