# Supplementary material for: An Aurora B-RPA signaling axis secures chromosome segregation fidelity
Source: Nat Commun. 2023 May 25;14:3008. doi: 10.1038/s41467-023-38711-2 (PMC10212944; doi:10.1038/s41467-023-38711-2)
Supplement: Supplementary file 4 — Source Data [file 41467_2023_38711_MOESM4_ESM.xlsx › Source Data II.pdf]

Figure 2.

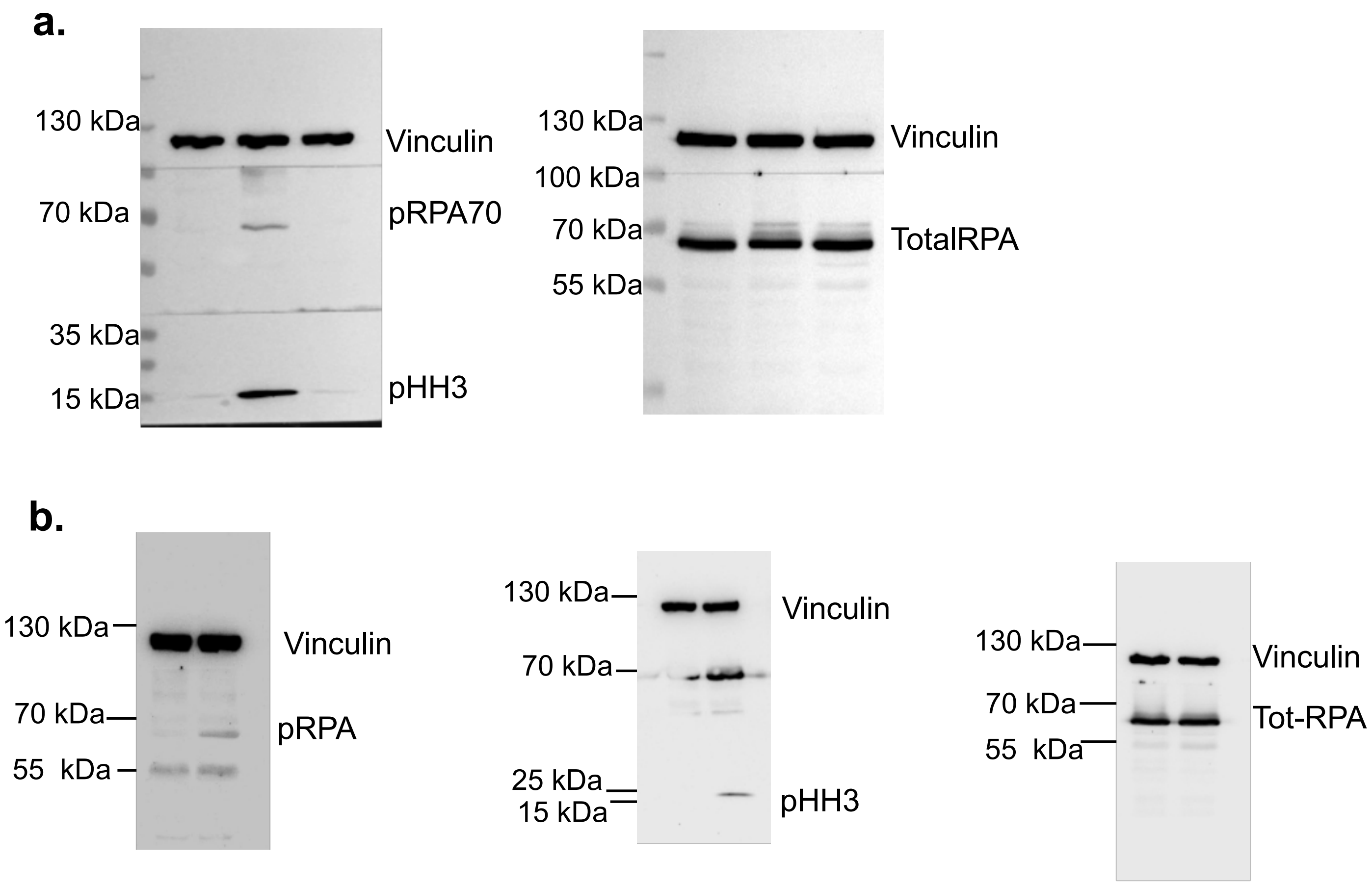
a.
130 kDa
Vinculin
70 kDa
pRPA70
35 kDa
15 kDa
pHH3
130 kDa
Vinculin
100 kDa
70 kDa
TotalRPA
55 kDa
b.
130 kDa
Vinculin
70 kDa
pRPA
55 kDa
130 kDa
Vinculin
70 kDa
25 kDa
15 kDa
pHH3
130 kDa
Vinculin
70 kDa
Tot-RPA
55 kDa

Figure 2.

c.

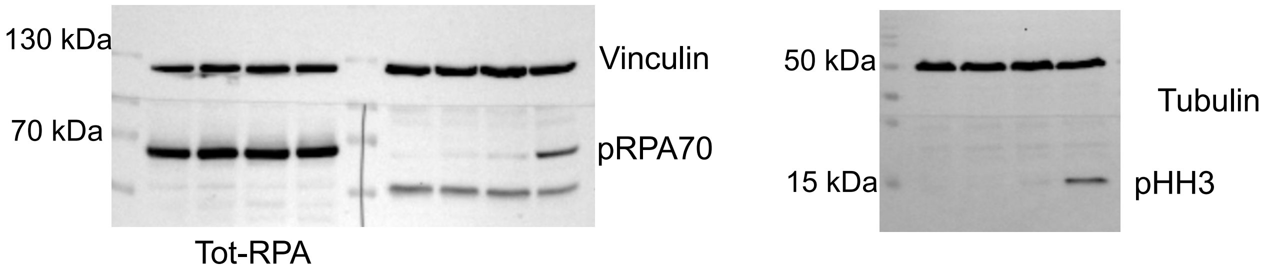
130 kDa
70 kDa
Vinculin
pRPA70
Tot-RPA
50 kDa
15 kDa
Tubulin
pHH3

e.

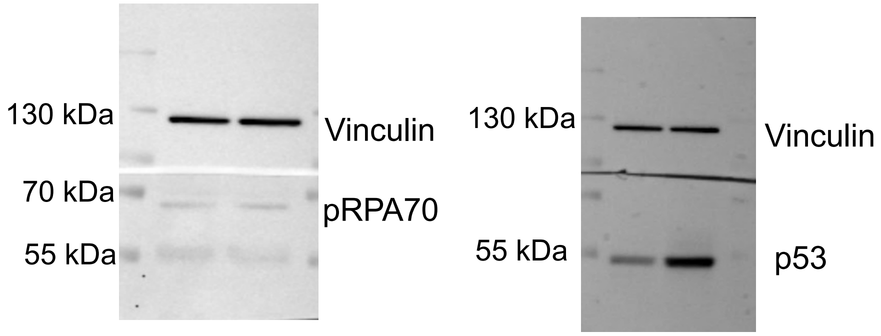
130 kDa
70 kDa
55 kDa
Vinculin
pRPA70
130 kDa
55 kDa
Vinculin
p53

d.

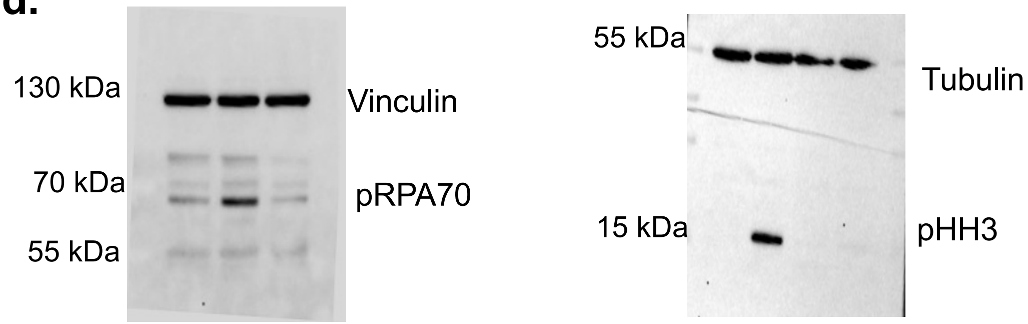
130 kDa
70 kDa
55 kDa
Vinculin
pRPA70
55 kDa
15 kDa
Tubulin
pHH3

Figure 4.

a.

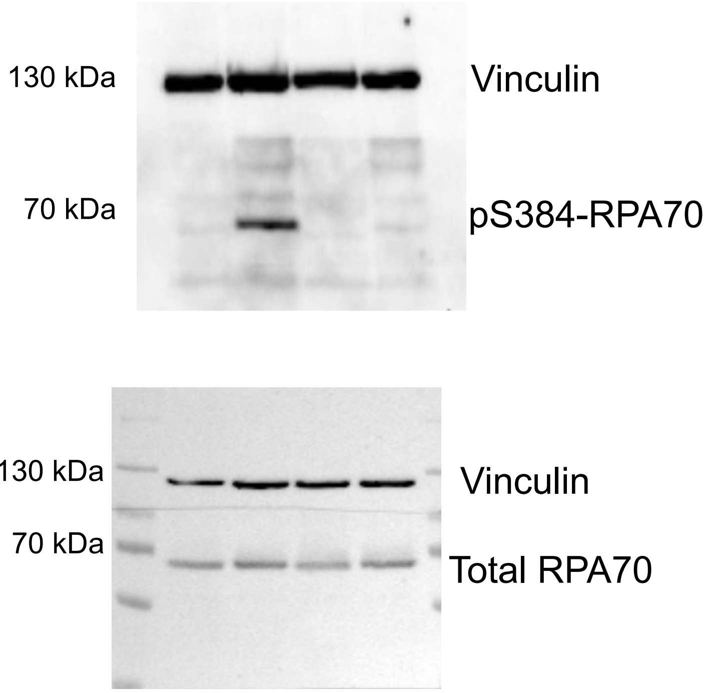
130 kDa
Vinculin
70 kDa
pS384-RPA70
130 kDa
Vinculin
70 kDa
Total RPA70

d.

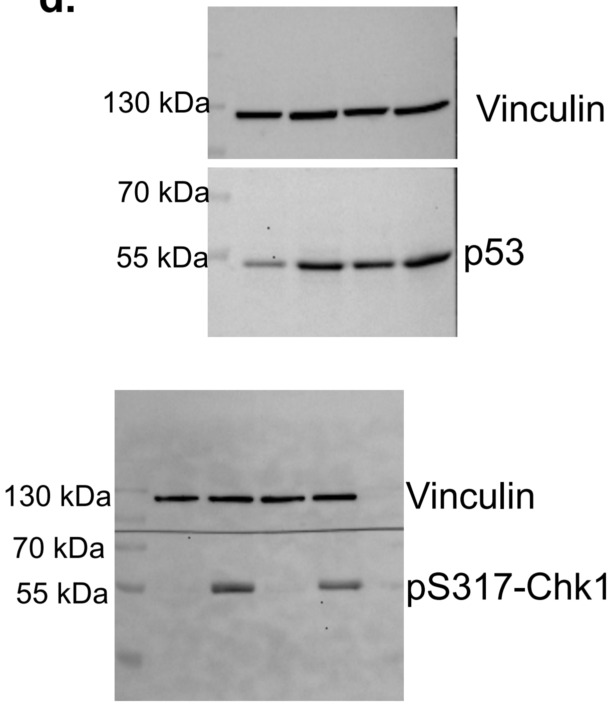
130 kDa
Vinculin
70 kDa
55 kDa
p53
130 kDa
Vinculin
70 kDa
55 kDa
pS317-Chk1

Figure 6.

a.

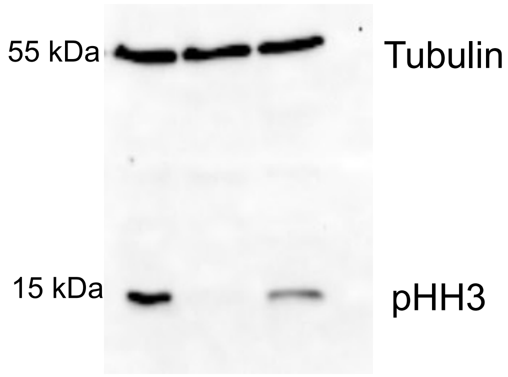
55 kDa
Tubulin
15 kDa
pHH3

c.

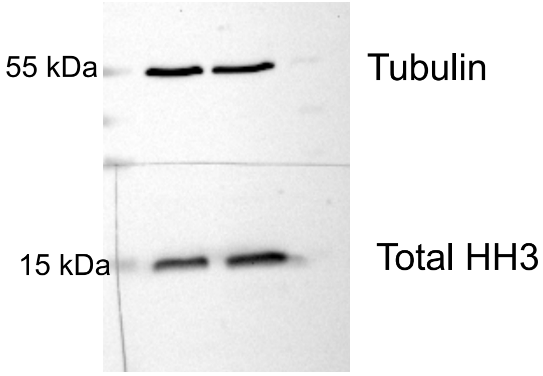
55 kDa
Tubulin
15 kDa
Total HH3

g.

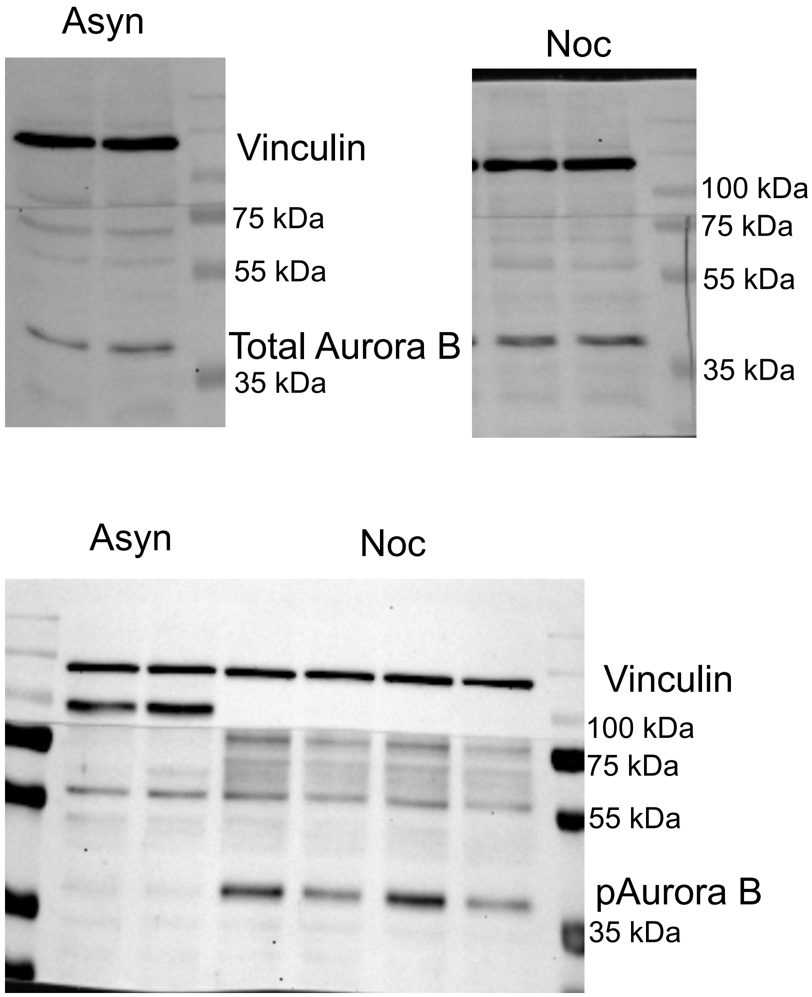
Asyn
Noc
Vinculin
75 kDa
55 kDa
Total Aurora B
35 kDa
100 kDa
75 kDa
55 kDa
35 kDa
Asyn
Noc
Vinculin
100 kDa
75 kDa
55 kDa
pAurora B
35 kDa

Suppl. Raw images

Figure S1 c.

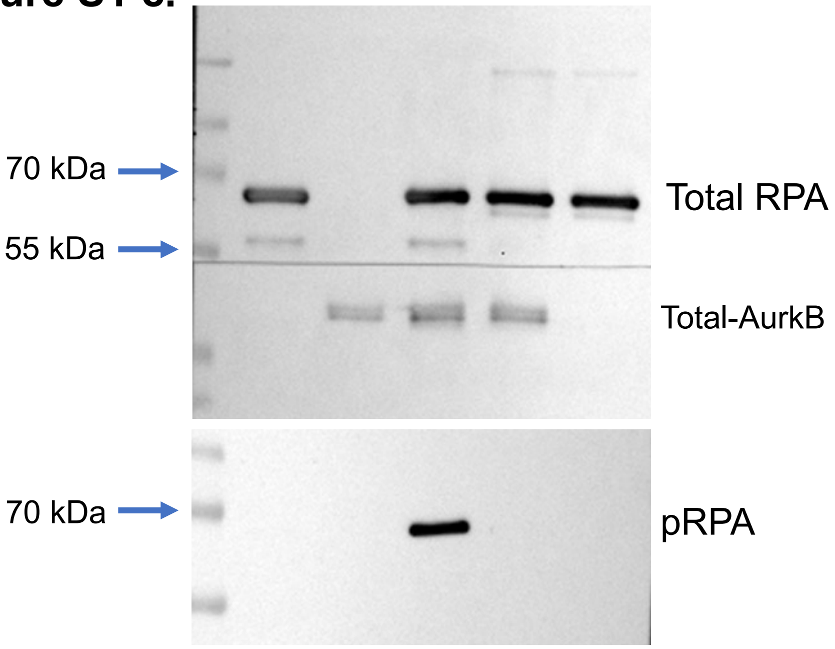

Figure S1 d.

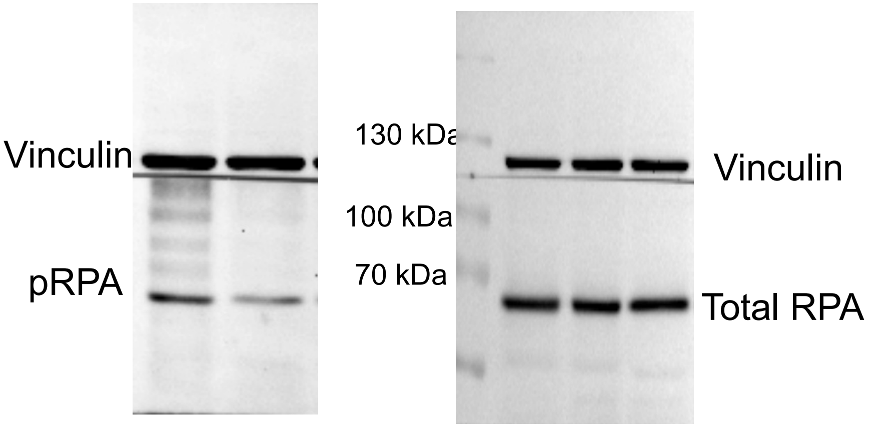

Figure S2.

e.

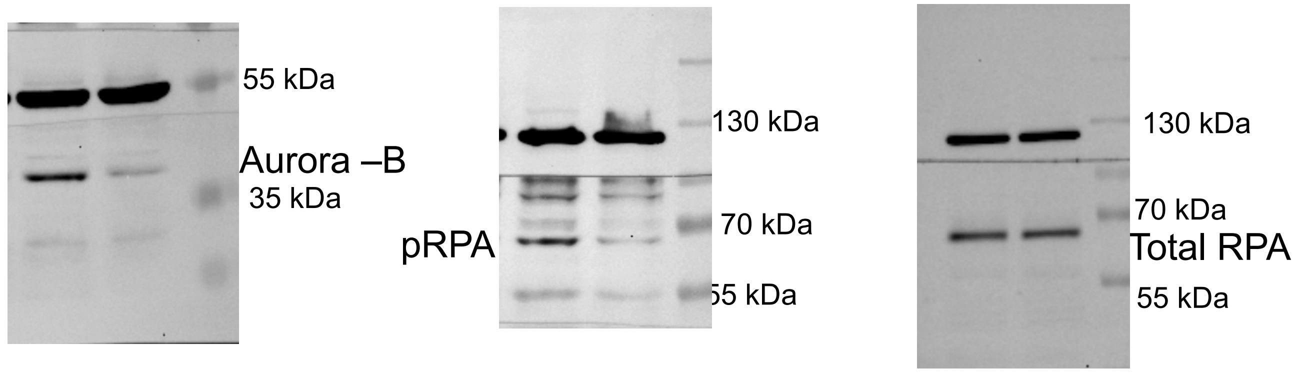

f.

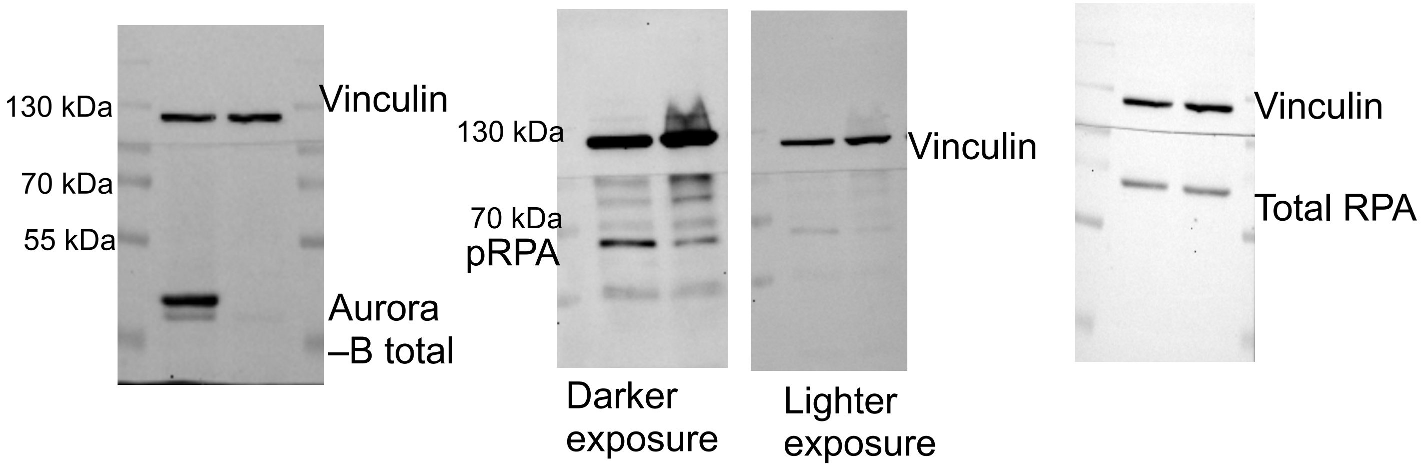

Figure S5 a.

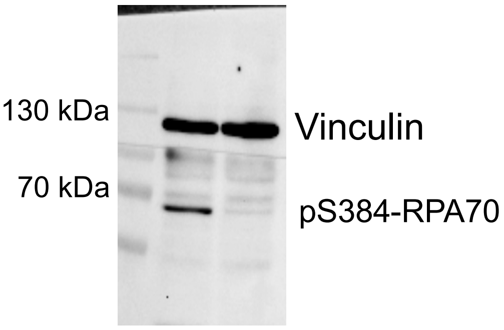

Figure S6 d.

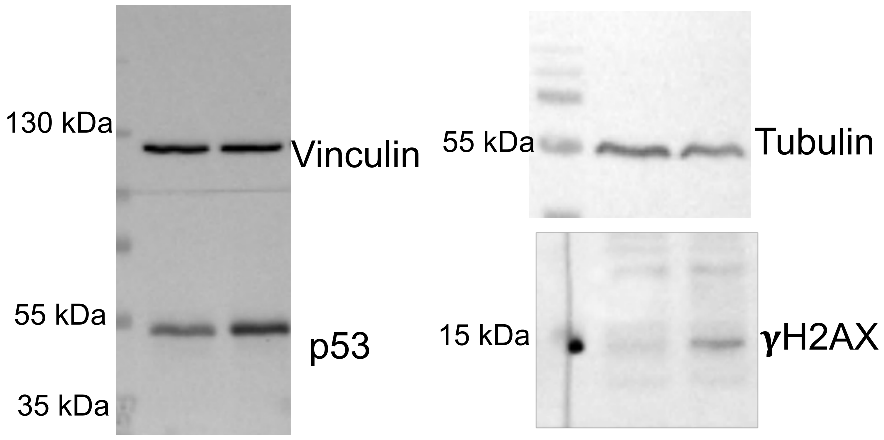

XCC216d_h_RPA1
the_Seq_start: GCTCTTTCCCCTAA
the_Seq_end: AGGGTTACTTGAGG
Test_Sequences:
sp2: GCCCGAGTCTCTGATTTCGGTGG
sp5: CCCGAGTCTCTGATTTCGGTGGA
R382Q: AGGAGCGCAAGTCTCT
S384A: CGAGTCGCTGATTT

GEIC-Plate36-C07 TOTAL:7200 OrderedDict([('sp2', 0), ('sp5', 0), ('R382Q', 0), ('S384A', 7095)])   [(0, 7180), (-1, 18), (-2, 2)]
GCTCTTTCCCCTAACTCTAGGAAAGCAGTGATAGTTTTGATGTGTCTCTGAGTAGATTCTCATGTGTGAG
GTCTGTCTTGTATGGATTCCATGTACGCTGATTACATTCACTCTACTATTGAATGTTTGCCTTGTTTTCT
ATAAAAATCTTCTGTGTTCTTCCTGCTAGTGACACTTGTATATGTCTGGTGTTTCTTTTACAGGCTGATA
AATTTGATGGTTCTAGACAGCCCGTGTTGGCTATCAAAGGAGCGCGAGTCGCTGATTTCGGTGGACGGAG
CCTCTCCGTGCTGTCTTCAAGCACTATCATTGCGAATCCTGACATCCCAGAGGCCTATAAGCTTCGTGGA
TGGTAGGTTTTGTGGGGCTAAACAAAGGGTTACT , 3008
GCTCTTTCCCCTAACTCTAGGAAAGCAGTGATAGTTTTGATGTGTCTCTGAGTAGATTCTCATGTGTGAG
GTCTGTCTTGTATGGATTCCATGTACGCTGATTACATTCACTCTACTATTGAATGTTTGCCTTGTTTTCT
ATAAAAATCTTCTGTGTTCTTCCTGCTAGTGACACTTGTATATGTCTGGTGTTTCTTTTACAGGCTGATA
AATTTGATGGTTCTAGACAGCCCGTGTTGGCTATCAAAGGAGCCCGAGTCGCTGATTTCGGTGGACGGAG
CCTCTCCGTGCTGTCTTCAAGCACTATCATTGCGAATCCTGACATCCCAGAGGCCTATAAGCTTCGTGGA
TGGTAGGTTTTGTGGGGCTAAACAAAGGGTTACT , 2902
GCTCTTTCCCCTAACTCTAGGAAAGCAGTGATAGTTTTGATGTGTCTCTGAGTAGATTCTCATGTGTGAG
GTCTGTCTTGTATGGATTCCATGTACGCTGATTACATTCACTCTACTATTGAATGTTTGCCTTGTTTTCT
ATAAAAATCTTCTGTGTTCTTCCTGCTAGTGACACTTGTATATGTCTGGTGTTTCTTTTACAGGCTGATA
AATTTGATGGTTCTAGACAGCCCGTGTTGGCTATCAAAGGAGCGCGAGTCGCTGATTTCGGTGGACGGAG
CCTCTCCATGCTGTCTTCAAGCACTATCATTGCGAATCCTGACATCCCAGAGGCCTATAAGCTTCGTGGA
TGGTAGGTTTTGTGGGGCTAAACAAAGGGTTACT , 123
GCTCTTTCCCCTAACTCTAGGAAAGCAGTGATAGTTTTGATGTGTCTCTGAGTAGATTCTCATGTGTGAG
GTCTGTCTTGTATGGATTCCATGTACGCTGATTACATTCACTCTACTATTGAATGTTTGCCTTGTTTTCT
ATAAAAATCTTCTGTGTTCTTCCTGCTAGTGACACTTGTATATGTCTGGTGTTTCTTTTACAGGCTGATA
AATTTGATGGTTCTAGACAGCCCGTGTTGGCTATCAAAGGAGCCCGAGTCGCTGATTTCGGTGGACGGAG
CCTCTCCATGCTGTCTTCAAGCACTATCATTGCGAATCCTGACATCCCAGAGGCCTATAAGCTTCGTGGA
TGGTAGGTTTTGTGGGGCTAAACAAAGGGTTACT , 119
GCTCTTTCCCCTAACTCTAGGAAAGCAGTGATAGTTTTGATGTGTCTCTGAGTAGATTCTCATGTGTGAG
GTCTGTCTTGTATGGATTCCATGTACGCTGATTACATTCACTCTACTATTGAATGTTTGCCTTGTTTTCT
ATAAAAATCTTCTGTGTTCTTCCTGCTAGTGACACTTGTATATGTCTGGTGTTTCTTTTACAGGCTGATA
AATTTGATGGTTCTAGACAGCCCGTGTTGGCTATCAAAGGAGCGCGAGTCGCTGATTTCGGTGGACGGAG
CCTCTCCGTGCTGTCTTCAATCACTATCATTGCGAATCCTGACATCCCAGAGGCCTATAAGCTTCGTGGA
TGGTAGGTTTTGTGGGGCTAAACAAAGGGTTACT , 27
GCTCTTTCCCCTAACTCTAGGAAAGCAGTGATAGTTTTGATGTGTCTCTGAGTAGATTCTCATGTGTGAG
GTCTGTCTTGTATGGATTCCATGTACGCTGAGTACATTCACTCTACTATTGAATGTTTGCCTTGTTTTCT
ATAAAAATCTTCTGTGTTCTTCCTGCTAGTGACACTTGTATATGTCTGGTGTTTCTTTTACAGGCTGATA
AATTTGATGGTTCTAGACAGCCCGTGTTGGCTATCAAAGGAGCGCGAGTCGCTGATTTCGGTGGACGGAG
CCTCTCCGTGCTGTCTTCAAGCACTATCATTGCGAATCCTGACATCCCAGAGGCCTATAAGCTTCGTGGA
TGGTAGGTTTTGTGGGGCTAAACAAAGGGTTACT , 22
GCTCTTTCCCCTAACTCTAGGAAAGCAGTGATAGTTTTGATGTGTCTCTGAGTAGATTCTCATGTGTGAG

GTCTGTCTTGTATGGATTCCATGTACGCTGAGTACATTCACTCTACTATTGAATGTTTGCCTTGTTTTCT
ATAAAAATCTTCTGTGTTCTTCCTGCTAGTGACACTTGTATATGTCTGGTGTTTCTTTTACAGGCTGATA
AATTTGATGGTTCTAGACAGCCCGTGTTGGCTATCAAAGGAGCCCGAGTCGCTGATTTCGGTGGACGGAG
CCTCTCCGTGCTGTCTTCAAGCACTATCATTGCGAATCCTGACATCCCAGAGGCCTATAAGCTTCGTGGA
TGGTAGGTTTTGTGGGGCTAAACAAAGGGTTACT , 20
GCTCTTTCCCCTAACTCTAGGAAAGCAGTGATAGTTTTGATGTGTCTCTGAGTAGATTCTCATGTGTGAG
GTCTGTCTTGTATGGATTCCATGTACGCTGATTACATTCACTCTACTATTGAATGTTTGCCTTGTTTTCT
ATAAAAATCTTCTGTGTTCTTCCTGCTAGTGACACTTGTATATGTCTGGTGTTTCTTTTACAGGCTGATA
AATTTGATGGTTCTAGACAGCCCGTGTTGGCTATCAAAGGAGCCCGAGTCGCTGATTTCGGTGGACGGAG
CCTCTCCGTGCTGTCTTCAATCACTATCATTGCGAATCCTGACATCCCAGAGGCCTATAAGCTTCGTGGA
TGGTAGGTTTTGTGGGGCTAAACAAAGGGTTACT , 16
GCTCTTTCCCCTAACTCTAGGAAAGCAGTGATAGTTTTGATGTGTCTCTGAGTAGATTCTCATGTGTGAG
GTCTGTCTTGTATGGATTCCATGTACGCTGATTACATTCACTCTACTATTGAATGTTTGCCTTGTTTTCT
ATAAAAATCTTCTGTGTTCTTCCTGCTAGTGACACTTGTATATGTCTGGTGTTTCTTTTACAGGCTGATA
AATTTGATGGTTCTAGACAGCCCGTGTTGGCTATCAAAGGAGCCCGAGTCGCTGATTTCGGTGGACGGAG
CCTCTCCGTGCTGTCTTCAACCACTATCATTGCGAATCCTGACATCCCAGAGGCCTATAAGCTTCGTGGA
TGGTAGGTTTTGTGGGGCTAAACAAAGGGTTACT , 12
GCTCTTTCCCCTAACTCTAGGAAAGCAGTGATAGTTTTGATGTGTCTCTGAGTAGATTCTCATGTGTGAG
GTCTGTCTTGTATGGATTCCATGTACGCTGATTACATTCACTCTACTATTGAATGTTTGCCTTGTTTTCT
ATAAAAATCTTCTGTGTTCTTCCTGCTAGTGACACTTGTATATGTCTGGTGTTTCTTTTACAGGCTGATA
AATTTGATGGTTCTAGACAGCCCGTGTTGGCTATCAAAGGAGCGTGAGTCGCTGATTTCGGTGGACGGAG
CCTCTCCGTGCTGTCTTCAAGCACTATCATTGCGAATCCTGACATCCCAGAGGCCTATAAGCTTCGTGGA
TGGTAGGTTTTGTGGGGCTAAACAAAGGGTTACT , 11
GCTCTTTCCCCTAACTCTAGGAAAGCAGTGATAGTTTTGATGTGTCTCTGAGTAGATTCTCATGTGTGAG
GTCTGTCTTGTATGGATTCCATGTACGCTGAATACATTCACTCTACTATTGAATGTTTGCCTTGTTTTCT
ATAAAAATCTTCTGTGTTCTTCCTGCTAGTGACACTTGTATATGTCTGGTGTTTCTTTTACAGGCTGATA
AATTTGATGGTTCTAGACAGCCCGTGTTGGCTATCAAAGGAGCGCGAGTCGCTGATTTCGGTGGACGGAG
CCTCTCCGTGCTGTCTTCAAGCACTATCATTGCGAATCCTGACATCCCAGAGGCCTATAAGCTTCGTGGA
TGGTAGGTTTTGTGGGGCTAAACAAAGGGTTACT , 11
GCTCTTTCCCCTAACTCTAGGAAAGCAGTGATAGTTTTGATGTGTCTCTGAGTAGATTCTCATGTGTGAG
GTCTGTCTTGTATGGATTCCATGTACGCTGATTACATTCACTCTACTATTGAATGTTTGCCTTGTTTTCT
ATAAAAATCTTCTGTGTTCTTCCTGCTAGTGACACTTGTATATGTCTGGTGTTTCTTTTACAGGCTGATA
AATTTGATGGTTCTAGACAGCCCGTGTTGGCTATCAAAGGAGCCTGAGTCGCTGATTTCGGTGGACGGAG
CCTCTCCGTGCTGTCTTCAAGCACTATCATTGCGAATCCTGACATCCCAGAGGCCTATAAGCTTCGTGGA
TGGTAGGTTTTGTGGGGCTAAACAAAGGGTTACT , 10

GEIC-Plate36-C11 TOTAL:6743 OrderedDict([('sp2', 0), ('sp5', 0), ('R382Q', 0), ('S384A', 6560)]) [(0, 6720), (-1, 20), (1, 1), (-2, 1), (-3, 1)]
GCTCTTTCCCCTAACTCTAGGAAAGCAGTGATAGTTTTGATGTGTCTCTGAGTAGATTCTCATGTGTGAG
GTCTGTCTTGTATGGATTCCATGTACGCTGATTACATTCACTCTACTATTGAATGTTTGCCTTGTTTTCT
ATAAAAATCTTCTGTGTTCTTCCTGCTAGTGACACTTGTATATGTCTGGTGTTTCTTTTACAGGCTGATA
AATTTGATGGTTCTAGACAGCCCGTGTTGGCTATCAAAGGAGCGCGAGTCGCTGATTTCGGTGGACGGAG
CCTCTCCGTGCTGTCTTCAAGCACTATCATTGCGAATCCTGACATCCCAGAGGCCTATAAGCTTCGTGGA
TGGTAGGTTTTGTGGGGCTAAACAAAGGGTTACT , 5266
GCTCTTTCCCCTAACTCTAGGAAAGCAGTGATAGTTTTGATGTGTCTCTGAGTAGATTCTCATGTGTGAG
GTCTGTCTTGTATGGATTCCATGTACGCTGATTACATTCACTCTACTATTGAATGTTTGCCTTGTTTTCT

ATAAAAATCTTCTGTGTTCTTCCTGCTAGTGACACTTGTATATGTCTGGTGTTTCTTTTACAGGCTGATA
AATTTGATGGTTCTAGACAGCCCGTGTTGGCTATCAAAGGAGCGCGAGTCGCTGATTTCGGTGGACGGAG
CCTCTCCATGCTGTCTTCAAGCACTATCATTGCGAATCCTGACATCCCAGAGGCCTATAAGCTTCGTGGA
TGGTAGGTTTTGTGGGGCTAAACAAAGGGTTACT , 198
GCTCTTTCCCCTAACTCTAGGAAAGCAGTGATAGTTTTGATGTGTCTCTGAGTAGATTCTCATGTGTGAG
GTCTGTCTTGTATGGATTCCATGTACGCTGATTACATTCACTCTACTATTGAATGTTTGCCTTGTTTTCT
ATAAAAATCTTCTGTGTTCTTCCTGCTAGTGACACTTGTATATGTCTGGTGTTTCTTTTACAGGCTGATA
AATTTGATGGTTCTAGACAGCCCGTGTTGGCTATCAAAGGAGCGCGAGTCGCTGATTTCGGTGGACGGAG
CCTCTCCGTGCTGTCTTCAATCACTATCATTGCGAATCCTGACATCCCAGAGGCCTATAAGCTTCGTGGA
TGGTAGGTTTTGTGGGGCTAAACAAAGGGTTACT , 39
GCTCTTTCCCCTAACTCTAGGAAAGCAGTGATAGTTTTGATGTGTCTCTGAGTAGATTCTCATGTGTGAG
GTCTGTCTTGTATGGATTCCATGTACGCTGAGTACATTCACTCTACTATTGAATGTTTGCCTTGTTTTCT
ATAAAAATCTTCTGTGTTCTTCCTGCTAGTGACACTTGTATATGTCTGGTGTTTCTTTTACAGGCTGATA
AATTTGATGGTTCTAGACAGCCCGTGTTGGCTATCAAAGGAGCGCGAGTCGCTGATTTCGGTGGACGGAG
CCTCTCCGTGCTGTCTTCAAGCACTATCATTGCGAATCCTGACATCCCAGAGGCCTATAAGCTTCGTGGA
TGGTAGGTTTTGTGGGGCTAAACAAAGGGTTACT , 31
GCTCTTTCCCCTAACTCTAGGAAAGCAGTGATAGTTTTGATGTGTCTCTGAGTAGATTCTCATGTGTGAG
GTCTGTCTTGTATGGATTCCATGTACGCTGAATACATTCACTCTACTATTGAATGTTTGCCTTGTTTTCT
ATAAAAATCTTCTGTGTTCTTCCTGCTAGTGACACTTGTATATGTCTGGTGTTTCTTTTACAGGCTGATA
AATTTGATGGTTCTAGACAGCCCGTGTTGGCTATCAAAGGAGCGCGAGTCGCTGATTTCGGTGGACGGAG
CCTCTCCGTGCTGTCTTCAAGCACTATCATTGCGAATCCTGACATCCCAGAGGCCTATAAGCTTCGTGGA
TGGTAGGTTTTGTGGGGCTAAACAAAGGGTTACT , 18
GCTCTTTCCCCTAACTCTAGGAAAGCAGTGATAGTTTTGATGTGTCTCTGAGTAGATTCTCATGTGTGAG
GTCTGTCTTGTATGGATTCCATGTACGCTGATTACATTCACTCTACTATTGAATGTTTGCCTTGTTTTCT
ATAAAAATCTTCTGTGTTCTTCCTGCTAGTGACACTTGTATATGTCTGGTGTTTCTTTTACAGGCTGATA
AATTTGATGGTTCTAGACAGCCCGTGTTGGCTATCAAAGGAGCGCGAGTCGCTGATTTCGGTGGACGGAG
CCTCTCCGTGCTGTCTTCAAGCACTATCATTGCGAATCCTGACATCCCAGAGGCCTATAAGCTTCGTGGA
TGGTAGGTTGTGTGGGGCTAAACAAAGGGTTACT , 16
GCTCTTTCCCCTAACTCTAGGAAAGCAGTGATAGTTTTGATGTGTCTCTGAGTAGATTCTCATGTGTGAG
GTCTGTCTTGTATGGATTCCATGTACGCTGATTACATTCACTCTACTATTGAATGTTTGCCTTGTTTTCT
ATAAAAATCTTCTGTGTTCTTCCTGCTAGTGACACTTGTATATGTCTGGTGTTTCTTTTACAGGCTGATA
AATTTGATGGTTCTAGACAGCCCGTGTTGGCTATCAAAGGAGCGCGAGTCGCTGATTTCGGTGGACGGAG
CCTCTCCGTGCTGTCTTCAACCACTATCATTGCGAATCCTGACATCCCAGAGGCCTATAAGCTTCGTGGA
TGGTAGGTTTTGTGGGGCTAAACAAAGGGTTACT , 16
GCTCTTTCCCCTAACTCTAGGAAAGCAGTGATAGTTTTGATGTGTCTCTGAGTAGATTCTCATGTGTGAG
GTCTGTCTTGTATGGATTCCATGTACGCTGATTACATTCACTCTACTATTGAATGTTTGCCATGTTTTCT
ATAAAAATCTTCTGTGTTCTTCCTGCTAGTGACACTTGTATATGTCTGGTGTTTCTTTTACAGGCTGATA
AATTTGATGGTTCTAGACAGCCCGTGTTGGCTATCAAAGGAGCGCGAGTCGCTGATTTCGGTGGACGGAG
CCTCTCCGTGCTGTCTTCAAGCACTATCATTGCGAATCCTGACATCCCAGAGGCCTATAAGCTTCGTGGA
TGGTAGGTTTTGTGGGGCTAAACAAAGGGTTACT , 14
GCTCTTTCCCCTAACTCTAGGAAAGCAGTGATAGTTTTGATGTGTCTCTGAGTAGATTCTCATGTGTGAG
GTCTGTCTTGTATGGATTCCATGTACGCTGATTACATTCACTCTACTATTGAATGTTTGCCTTGTTTTCT
ATAAAAATCTTCTGTGTTCTTCCTGCTAGTGACACTTGTATATGTCTGGTGTTTCTTTTACAGGCTGATA
AATTTGATGGTTCTAGACAGCCCGTGTTGGCTATCAAAGGAGCGCGAGTCGCTGATTTCGGTGGACGGAG
CCTCTCCGTGCTGTCTTCAAACACTATCATTGCGAATCCTGACATCCCAGAGGCCTATAAGCTTCGTGGA
TGGTAGGTTTTGTGGGGCTAAACAAAGGGTTACT , 13
GCTCTTTCCCCTAACTCTAGGAAAGCAGTGATAGTTTTGATGTGTCTCTGAGTAGATTCTCATGTGTGAG
GTCTGTCTTGTATGGATTCCATGTACGCTGATTACATTCACTCTACTATTGAATGTTTGCCTTGTTTTCT
ATAAAAATCTTCTGTGTTCTTCCTGCTAGTGACACTTGTATATGTCTGGTGTTTCTTTTACAGGCTGATA
AATTTGATGGTTCTAGACAGCCCGTGTTGGCTATCAAAGGAGCGTGAGTCGCTGATTTCGGTGGACGGAG

CCTCTCCGTGCTGTCTTCAAGCACTATCATTGCGAATCCTGACATCCCAGAGGCCTATAAGCTTCGTGGA
TGGTAGGTTTTGTGGGGCTAAACAAAGGGTTACT , 12
GCTCTTTCCCCTAACTCTAGGAAAGCAGTGATAGTTTTGATGTGTCTCTGAGTAGATTCTCATGTGTGAG
GTCTGTCTTGTATGGATTCCATGTACGCTGATTACATTCACTCTACTATTGAATGTTTGCCTTGTTTTCT
ATAAAAATCTTCTGTGTTCTTCCTGCTAGTGACACTTGTATATGTCTGGTGTTTCTTTTACAGGCTGATA
AATTTGATGGTTCTAGACAGCCCGTGTTGGCTATCAAAGGAGCGCGAGTCGCTGATTTCGGTGGACGGAG
CCTCTCCGTGCTGTCTTCAAGCGCTATCATTGCGAATCCTGACATCCCAGAGGCCTATAAGCTTCGTGGA
TGGTAGGTTTTGTGGGGCTAAACAAAGGGTTACT , 11
GCTCTTTCCCCTAACTCTAGGAAAGCAGTGATAGTTTTGATGTGTCTCTGAGTAGATTCTCATGTGTGAG
GTCTGTCTTGTATGGATTCCATGTACGCTGATTACATTCACTCTACTATTGAATGTTTGCCGTGTTTTCT
ATAAAAATCTTCTGTGTTCTTCCTGCTAGTGACACTTGTATATGTCTGGTGTTTCTTTTACAGGCTGATA
AATTTGATGGTTCTAGACAGCCCGTGTTGGCTATCAAAGGAGCGCGAGTCGCTGATTTCGGTGGACGGAG
CCTCTCCGTGCTGTCTTCAAGCACTATCATTGCGAATCCTGACATCCCAGAGGCCTATAAGCTTCGTGGA
TGGTAGGTTTTGTGGGGCTAAACAAAGGGTTACT , 10
